# Faster life history strategy manifests itself by lower age at menarche, higher sexual desire, and earlier reproduction in people with worse health

**DOI:** 10.1038/s41598-021-90579-8

**Published:** 2021-05-27

**Authors:** Kateřina Sýkorová, Jaroslav Flegr

**Affiliations:** 1 Department of Philosophy and History of Science, Faculty of Science, Charles University, Vinicna 7, 128 00 Prague, Czech Republic; 2 Department of Applied Neurosciences and Brain Imagination, National Institute of Mental Health, Topolova 748, Klecany, Czech Republic

**Keywords:** Ecology, Evolution

## Abstract

Factors which indicate lower life expectancy also induce switching to a faster life strategy, that is, a higher investment in current reproduction at the expense of future reproduction and body maintenance. We tested a hypothesis according to which impairment of individual health serves as a signal for switching to a faster life strategy using online-gathered data from 32,911 subjects. Worse health was associated with lower age at menarche and earlier initiation of sexual life in women and higher sexual desire and earlier reproduction in both sexes. Individuals with worse health also exhibited lower sexual activity, lower number of sexual partners, and lower total number of children. These results suggest that impaired health shifts individuals towards a faster life strategy but also has a negative (physiological) effect on behaviours related to sexual life. Signs of a faster life strategy were also found in Rh-negative men in good health, indicating that even just genetic predisposition to worse health could serve as a signal for switching to a faster life strategy. We suggest that improved public health in developed countries and the resulting shift to a slower life strategy could be the ultimate cause of the phenomenon of demographic transition.

## Introduction

Life history theory explains the allocation of organism’s resources to growth, body maintenance, and reproduction, thereby providing a framework for understanding the developmental and reproductive strategies of individuals, populations, and species. Life history theories predict that organisms adopt a faster life history strategy, i.e. a higher investment in current as opposed to future reproduction, in response to several environmental factors. These are, for example, factors which reduce adult life expectancy^1, 2^, low competition for resources in low population density^3, 4^, or top-down (turbidostat-like) type of regulation of population size^5, 6^. Adoption of a particular life strategy takes place on both a species and population level by shifting the frequencies of corresponding alleles^7, 8^. On an individual level, it manifests itself as an adaptive modification of resource allocation or behaviour depending on current situation^9–12^.

Most modern human populations are characterised by low morbidity and mortality and a high population density^1^. It is widely accepted that such overcrowded and low-mortality environments lead to increased competitiveness in human societies, thus favouring so-called ‘K-strategists’, that is, individuals who invest more biological and material resources into a lower number of highly competitive offspring^3, 4^. The opposite strategy is followed by ‘r-strategists’, i.e. individuals who invest into more numerous but less competitive offspring. At the same time, low adult morbidity and mortality is also associated with high life expectancy, which favours another strategy, namely a slower life strategy characterised by higher investment in own development and postponement of reproduction to an optimal stage of life^1, 13–15^.

Characteristics expressed by faster- and r-strategists (or slower- and K-strategists) are in many situations identical. Under some conditions, however, these strategies can dramatically diverge. It is therefore crucial to distinguish between the r/K and fast/slow theories. A switch from a K- to an r-strategy follows after a reduction of population density and concurrent decrease of competition for resources. This transition is accompanied by an increased natality rate (grams of biomass/time) at the expense of production efficiency (grams of biomass produced/grams of resources consumed)^5, 6^. A shift from the slower to a faster life strategy, on the other hand, is driven by increased mortality, especially mortality of middle-aged adults^1, 14^. A reduction of population density may be the result of increased mortality in a population, in which case a shift from a K- to an r-strategy usually coincides with slow-to-fast strategy transition. If, however, reduction of population density is due to activity of a parasite or predator that preferentially kills (immunologically naive) juveniles or smaller-sized individuals, then increased pressure exerted by this parasite/predator (which reduces population density) will favour the slower life strategy regardless of the fact that the organisms are r- rather than K-strategists. When, on the other hand, a parasite/predator preferentially kills or disables adult members of a population, the increased pressure it exerts will result in the adoption of a faster life strategy, which prioritises current over future reproduction.

It ought to be mentioned that life history theories came to be understood differently in different disciplines, especially in the past fifteen years^16^. Some researchers view the r/K theory as not entirely distinct from fast/slow concept: they see it rather as an older and simplistic version of it and currently even abandoned and supplanted by fast/slow terminology^16^. Others, though, differentiate between the r/K framework based on density-dependent selection and models focusing on age-dependent survival and mortality^17^.

Life strategies developed in response to various environmental conditions is a subject examined by numerous studies. People exposed to harsh or unpredictable environment, such as is usually attendant upon low socioeconomic status, dangerous environment during development, or unfavourable family circumstances, tend to adopt faster life strategies. They start their sexual life earlier^18^ and women have lower age at menarche and first birth and have more children^19–23^.

Belsky et al.^24^ in their influential article proposed that individuals use the environment experienced during childhood as a forecast of future environment and therefore develop reproductive strategies corresponding to those early conditions. Rickard et al.^25^ recently contrasted this explanation with their proposal, the ‘internal prediction model’, according to which stress experienced in adverse childhood damages individuals’ bodies, which leads to worse expected adult health and shortens likely reproductive period. Individuals then adaptively respond to their impaired internal states by adoption of a faster life strategy. The results of Chua et al.^26^ suggest that people develop life strategies in response to both cues of the external early environment and internal conditions of bodies.

The internal prediction model assumes that internal state works as an indicator of exposure to harsh environment during childhood (or the ability to withstand such harshness). From this perspective, current individual health should form the foundation of more reliable forecast of future health and lifespan than a mere observation of environmental harshness and unpredictability^26^. As such, it should thus have a more direct impact on the profitability of particular life strategies. Despite these considerations, much less effort has been invested in studying the effects of individual health status on the probability of switching to a faster life strategy. It is evident that if an individual suffers from a severe illness—or possibly a combination of several less severe health impairments—that cannot be overcome by redirection of more resources to body maintenance, the expected time available for reproduction will be reduced. For such individuals, immediate reproduction, instead of postponement of reproduction to an optimal period of life, should be the most adaptive strategy.

The effect of individual health on the shift from a slower to a faster life strategy has been tested mostly in animal species ranging from insects to mammals. For example, an experimental parasite infection led to higher investment in reproduction of snails, fruit flies, crickets, ants, and deer mice^9, 10, 12, 27, 28^. The results of such experiments, as well as of observational studies^29–31^, show that health impairment and related lower likelihood of later reproduction might induce switching to a faster life strategy in various animal species.

The impact of health on human life strategies had so far received much less attention. Westendorp and Kirkwood^32^ and Thomas et al.^33^ demonstrated that in women, longevity negatively correlates with the number of children and positively with the age at their first birth. Women who live longer were probably healthier and could therefore be expected to adopt a slower life strategy. They could postpone reproduction to an optimal age, which automatically results in having fewer children of higher quality. The authors of these studies, however, suggested the opposite causality for the observed association between health and slower life strategy. They viewed the lower reproduction rate as the cause of better health and longevity in women, while we suggest that in women, good health leads to a lower rate of reproduction. Other studies found positive correlations between self-rated health and behaviour associated with a slower life strategy, such as quality of relationships, amount of social contact and support, altruism, anticipation, insight into the past, planning, persistence, ability to delay gratification, low impulsiveness, long-term mating, and mental health^26, 34–36^.

Uggla and Mace^37^ observed an association between the birth rate in Northern Irish women under the age of 23 and morbidity rate in their place of residence. On the other hand, ward-level morbidity, i.e. morbidity within a small, well-defined area, is just a very rough proxy for an individual’s impaired health. What is important, is that the effect disappeared once the value of an individual’s house was controlled for. The authors therefore suggested that family’s economic situation is a better predictor of low age at first birth than morbidity rates are. So far probably the most important study providing direct support for the hypothesis of impaired health inducing a faster life strategy in humans was published by Waynforth^38^. In his longitudinal study, he followed British children diagnosed in childhood with a chronic disease such as epilepsy, type 1 diabetes, asthma, or congenital heart defects, and found they were 1.6 times more likely to have had their first child by the age of thirty. In accordance with predictions of the life strategy hypothesis, Waynforth considered impaired individual health to be a factor that reduces one’s life expectancy and thereby induces an appropriate compensatory response in the timing of reproduction. Most recently, Chua et al.^26^ used structural equation modelling to search for direct and health-mediated effects of harsh environments on traits related to the faster life strategy and they found a direct positive effect of self-reported worse health on the number of children.

Pressure on maximal competitiveness of offspring, which leads to the adoption of K-strategy, is often viewed as the ultimate cause of a demographic transition in the form of a dramatic decrease of fecundity in modern developed countries^39–41^. Higher living standards, the advent of modern medicine, and practical elimination of starvation also led to an improvement in the general health of most inhabitants of developed countries^42^. This improved general health consequently led to a dramatic increase in mean life expectancy. We suggest that better individual health and higher likelihood of future reproduction increased the biological profitability of a slower life strategy while impaired individual health, due for instance to infectious diseases or genetic predispositions, increased the profitability of faster life strategy. People in poor health should reproduce as early as possible, that is, they should reproduce before their worsening health diminishes their chances of any reproduction. In contrast, people in better health can postpone their reproduction to an optimal age, i.e. a time when they accumulate sufficient physiological, material, and social resources, but are still physiologically well capable of reproduction. Adoption of a particular life strategy is, of course, only rarely the result of rational analysis and decision.

The abovementioned demographic transition could be, therefore, a consequence of either switching from an r- to K-strategy in an overcrowded and highly competitive environment (as is mostly believed) or, alternatively, the result of switching from a faster to a slower life strategy in populations where most people are healthy and consequently have a high life expectancy. These two explanations of the demographic transition differ in their general predictions. The fast/slow theory predicts that individuals with a lower life expectancy, e.g. those who suffer from worse health or are predisposed to some disease, will start reproducing earlier than individuals with a higher life expectancy. The r/K theory, on the other hand, predicts that less healthy individuals will start reproducing later because it will take them longer to accumulate sufficient physiological and material resources to ensure competitiveness of their offspring. To decide which of these two alternative explanations of the demographic transition is valid, one must be in possession of data on the effect of individual health on life history parameters.

The important issue for any observational study is the problem of causality. It is not readily apparent whether an individual’s faster life strategy is the result of their worse health or whether their worse health is the effect of their faster (and riskier) life strategy. Optimal approach to this conundrum would involve examination of association between the faster life strategy with not only worse health but also some genetic predisposition to worse health. In the Czech population, Rh-negative genotype might serve as such predictor. Five previous studies had shown associations between Rh-negativity and psychomotor or cognitive functions^43–47^ and other four studies had demonstrated the existence of a relation between Rh-negativity and health^48–51^. Another large-scale study concluded that Rh-negative mothers give birth to significantly more girls than boys: secondary sex ratio 1.37 in primiparous mothers and 1.23 in all mothers^52^. According to the Trivers-Willard hypothesis, this indicates worse health of Rh-negative women. These studies suggest that Rh-negativity, with its negative impact on health, is maintained in human populations due to Rh-positive heterozygotes’ higher resistance to certain diseases by selection in favour of heterozygotes. To the best of our knowledge, only two published studies found no effect of Rh-negativity on human performance and mortality^53, 54^. The problem of Rh-negativity is that its effect on health is relatively small. This is at least in part because existing studies for the most part compared the health of Rh-negatives with the health of a mixed population consisting of both Rh-positive heterozygotes (who enjoy better health) and Rh-positive homozygotes, who in some respects suffer from even worse health than Rh-negative subjects^51^. On the other hand, the main advantage of Rh factor is that a large fraction of Czech respondents can report their Rh blood group in questionnaire studies.

In the present cross-sectional study, we tested whether impaired health serves as a signal for switching to a faster life strategy in humans. In doing so, we tested validity of the main assumption of hypothesis regarding the shift from fast to slow life strategy in context of the demographic transition. In particular, we examined whether impaired health is associated with the following signs of the faster life strategy: lower age at menarche, lower age at first sexual intercourse, higher sexual desire, higher sexual activity, higher number of sexual partners, and earlier reproduction. To address the subjectivity of respondents’ self-reported health and to control for the issue of possible opposite causality, we looked in our analyses at genetic predisposition to worse health in the form of Rh-negative genotype instead of worse health as such as a predictor of adoption of faster life strategy.

To test thirteen specific hypotheses related to our general prediction (see Table 1), we collected anonymous responses about participants’ health and behaviours related to sexual life and reproduction from 32,911 subjects. The study, including all thirteen specific hypotheses to be tested, the stopping rule and methods of statistical analyses, was preregistered prior to its commencement as ‘Relation between a health condition and life-history traits in humans’ on the website of the Open Science Framework (https://osf.io/wfbkr/).

**Table 1:** Thirteen hypotheses tested in the confirmatory part of the study and their confirmation in GLM or path analyses. Hypotheses consistent with the results of our preregistered data analyses are marked by ‘**’; and hypothesis consistent with the results of our data analyses in the exploratory part is marked by ‘*’.

h1**	Age at menarche in less healthy women is lower than in other women
h2*	Less healthy women initiate sexual life earlier than other women
h3**	Less healthy women exhibit higher sexual desire than other women
h4	Less healthy women are more sexually active (report more sexual intercourse) than other women
h5	Less healthy women are more promiscuous (report more sexual partners) than other women
h6**	Less healthy women have more children earlier in life and fewer children later in life than other women
h7	Rh-negative women have more children earlier in life and fewer children later in life than Rh-positive women
h8	Less healthy men initiate sexual life earlier than other men
h9**	Less healthy men exhibit higher sexual desire than other men
h10	Less healthy men are more sexually active (report more sexual intercourse) than other men
h11	Less healthy men are more promiscuous (report more sexual partners) than other men
h12**	Less healthy men have more children earlier in life and fewer children later in life than other men
h13	Rh-negative men have more children earlier in life and fewer children later in life than Rh-positive men

## Results

### Descriptive statistics

The first set contained the data of 13,408 women and 16,419 men (mean age of women = 31.2, s.d. = 11.4; mean age of men = 35.8, s.d. = 12.5). The second dataset including at least some information about respondents’ health contained 16,634 women and 16,277 men with mean age 31.2 (s.d. = 11.4) and 35.8 (s.d. = 12.4), respectively. Descriptive statistics for all variables used in this study are presented in Supplementary Table S1. Cross-correlations between source variables of the sickness index are found in Supplementary Table S2.

All output variables significantly correlated with age (see Supplementary Table S3). In accordance with the preregistered protocol, we computed correlations between all output variables and potential covariates (socioeconomic status, current partnership, height, size of place of residence, education, church membership) using partial Kendall correlation test with age as a covariate (Supplementary Table S3).

### Confirmatory part of the study

Before embarking on the present study, we postulated (and preregistered) thirteen hypotheses (see Table 1). Table 1 also indicates which were supported by the results of our statistical tests.

The multivariate multiple GLM analysis with age at first sexual intercourse, sexual desire, sexual activity, number of sexual partners, number of children, and age at menarche in women as dependent variables and age, socioeconomic status, current partnership, height, size of place of residence, education, church membership, sickness index, and interaction between the sickness index and age as independent variables showed a significant effect of the sickness index on variables related to sexual life and reproduction in both women (p < 0.005, η^2^ = 0.011) and men (p < 0.005, η^2^ = 0.013).

**Figure 1:**
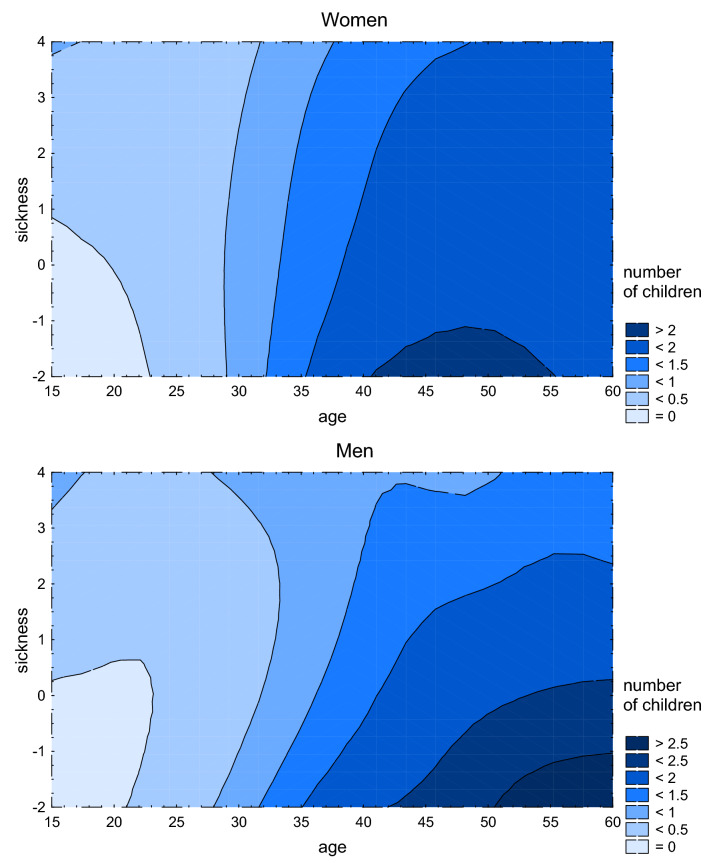
The effects of sickness index and age on the number of children in women and men. This figure shows XYZ contour plots with distance-weighted least-squares fitting used for extrapolation. If an interaction between the sickness index and age had no effect on the number of children, the differently coloured areas would form vertical stripes.

Subsequent eleven univariate GLM analyses (Table 2) showed a significant negative association of the sickness index with age at menarche in women and a significant positive association with sexual desire in both women and men. These results thus support hypotheses h1, h3, and h9. Interaction between the sickness index and age had a significant effect on the number of children in both women and men. Contour plots suggest that healthier people have fewer children before 25 years of age than less healthy people do (Fig. 1). Nevertheless, after the age of 40, healthier people tend to have more than two children while less healthy people still have fewer than two children. The existence and nature of this significant interaction between the sickness index and age are in agreement with our hypotheses h6 and h12.

**Table 2:** Associations of the sickness index and covariates with each variable related to sexual life and reproduction. This table shows the results (standardised beta coefficients) of eleven univariate GLM analyses (Type III sums of squares) analysing associations between variables related to sexual life and reproduction (first column) and the sickness index and covariates. In accordance with the preregistration, each model includes only covariates with Kendall’s Tau > 0.02 or < − 0.02 for correlation with output variables (see Supplementary Table S3). Current partnership and church membership were treated as binary variables with 0 indicating no current partnership and no church membership, and 1 indicating being in a partnership and church membership. A positive beta therefore means that for example subjects currently in a partnership score higher on a particular variable. Associations with p values under 0.05 are in bold and those with p values under 0.005 are marked with asterisks.

	Age	Socioeconomic status	Current partnership	Height	Size of place of residence	Education	Church membership	Sickness index	Sickness index–age interaction
**Women**									
Age at menarche	0.115*			0.116*		0.018		− 0.093*	− 0.013
Age at first sexual intercourse	0.122*		− 0.109*	0.028*		0.250*	0.339*	0.004	0.038
Sexual desire	− 0.155*	− 0.013	− 0.563*			0.003	− 0.190*	0.125*	− 0.056*
Sexual activity	− 0.165*	− 0.008	0.963*	0.025*				− 0.096*	− 0.098*
Number of sexual partners	− 0.110*		− **0.047**	0.033*	**0.027**		− 0.209*	− **0.044**	− 0.106*
Number of children	0.708*		0.286*	0.002		− 0.070*	0.128*	− 0.076*	− 0.077*
**Men**									
Age at first sexual intercourse	0.106*		− 0.135*		− 0.045*	0.207*	0.188*	0.062*	0.014
Sexual desire	− 0.121*		− 0.218*			0.012	− 0.099*	0.140*	− 0.054*
Sexual activity	− 0.090*	**0.019**	1.005*	0.023*			− 0.107*	− 0.158*	− **0.043**
Number of sexual partners	− 0.015	0.038*	0.211*	**0.020**	**0.017**		− 0.145*	− 0.058*	− 0.024
Number of children	0.628*	0.022*	0.422*		− 0.020*	− **0.014**	0.162*	− 0.041*	− 0.109*

GLM analyses also revealed a significant negative effect of the sickness index on sexual activity and the number of sexual partners in both women and men. These results opposed our hypotheses h4, h5, h10, and h11. In men, we observed a positive association between the sickness index and age at first sexual intercourse, which contradicts hypothesis h8. We detected neither an effect of interaction between the Rh phenotype and age (Table 3), nor an effect of interaction between Rh phenotype, the sickness index, and age (Supplementary Table S4) on the number of children. This contradicts our hypotheses h7 and h13 (but see results of the exploratory part below). All of the results of preregistered tests retained their significance after the Benjamini–Hochberg correction.

**Table 3:** Associations of Rh-negativity and covariates with each variable related to sexual life and reproduction. This table shows the results (standardised beta coefficients) of eleven univariate GLM analyses (Type III sums of squares) analysing associations between variables related to sexual life and reproduction (first column) and Rh-negativity and covariates. In accordance with the preregistration, each model includes only covariates with Kendall’s Tau > 0.02 or < − 0.02 for correlation with output variables (see Supplementary Table S3). Current partnership, church membership, and Rh-negativity were binary variables with 0 indicating no current partnership, no church membership, and a positive Rh phenotype, and 1 indicating being in a partnership, church membership, and a negative Rh phenotype. Associations with p values under 0.05 are in bold and those with p values under 0.005 are marked with asterisks.

	Age	Socioeconomic status	Current partnership	Height	Size of place of residence	Education	Church membership	Rh-negativity	Rh–age interaction
**Women**									
Age at menarche	0.130*			0.107*		**0.031**		0.028	− 0.037
Age at first sexual intercourse	0.105*		− 0.143*	**0.032**		0.240*	0.377*	0.007	0.011
Sexual desire	− 0.170*	− **0.001**	− 0.495*			− 0.016	− 0.168*	− 0.014	0.008
Sexual activity	− 0.174*	0.004	0.927*	0.018				− 0.022	− 0.032
Number of sexual partners	− 0.140*		− 0.001	0.024	0.017		− 0.197*	− 0.021	0.044
Number of children	0.676*		0.335*	0.007		− 0.053*	0.138*	0.012	0.032
**Men**									
Age at first sexual intercourse	0.108*		− 0.160*		− 0.046*	0.194*	0.248*	− **0.080**	− 0.047
Sexual desire	− 0.149*		− 0.244*			− 0.005	− 0.130*	0.025	**0.064**
Sexual activity	− 0.122*	0.032*	0.953*	0.007			− 0.127*	0.036	0.051
Number of sexual partners	− 0.026	0.041*	0.186*	0.017	0.017		− 0.202*	**0.073**	− 0.044
Number of children	0.590*	0.032*	0.492*		− 0.027*	− 0.001	0.158*	0.046	0.023

Interaction between the sickness index and age showed a significant association with sexual desire and sexual activity in both women and men and with the number of sexual partners in women (Table 2). Contour plots showed a higher sexual desire in less healthy women under 50 years of age and in less healthy men over 50 years of age (Supplementary Fig. S1). Both sexual activity in women and men and the number of sexual partners in women decreased more rapidly with age when they were less healthy (Supplementary Figs. S2–3). The existence and nature of these interactions support our main hypothesis about faster life strategy in less healthy people, but they were not part of the preregistered protocol.

Taken together, our results support five of the thirteen preregistered hypotheses. In the follow-up exploratory part of the study, we will explore the role of potentially mediating variables using path analysis and the effect of Rh-negativity, i.e. a genetic predisposition to worse health.

### Exploratory part of the study

Our preregistered GLM analyses did not allow us to take into account subjects who have not yet had menarche or first sexual intercourse. In theory, exclusion of these respondents from analyses could lead to biased results. In our set, menarche has not been reached only by nine (i.e. 0.09%) women, which cannot have affected the results. Nevertheless, 3.7% of women and 4.7% of men reported not having had their first sexual intercourse yet. We conducted the Cox regression to include the effect of respondents who have not yet started their sexual life. We found that the likelihood of having first sexual intercourse is independent of worse health in women (hazard ratio = 1.014, p = 0.502) but decreases with worse health in men (hazard ratio = 0.904, p < 0.005). In other words, less healthy men take more time to reach their first sexual intercourse. The results of Cox regression therefore correspond to the results of GLM for both women and men.

We found a significant negative relation between the sickness index and age at first sexual intercourse in women (Kendall’s Tau = − 0.036, p < 0.005) but once the set of covariates including education was entered into the statistical model, the association lost its significance (Table 2). The sickness index, on the other hand, showed a relatively (in comparison to other associations in the study) strong negative correlation with education (Kendall’s Tau = − 0.101, p < 0.005), which might indicate that education has a mediating role in this association. In essence, it thus seems that lower investment in education (and therefore lower investment in future) could be an important part of the faster life strategy of people with impaired health.

**Figure 2:**
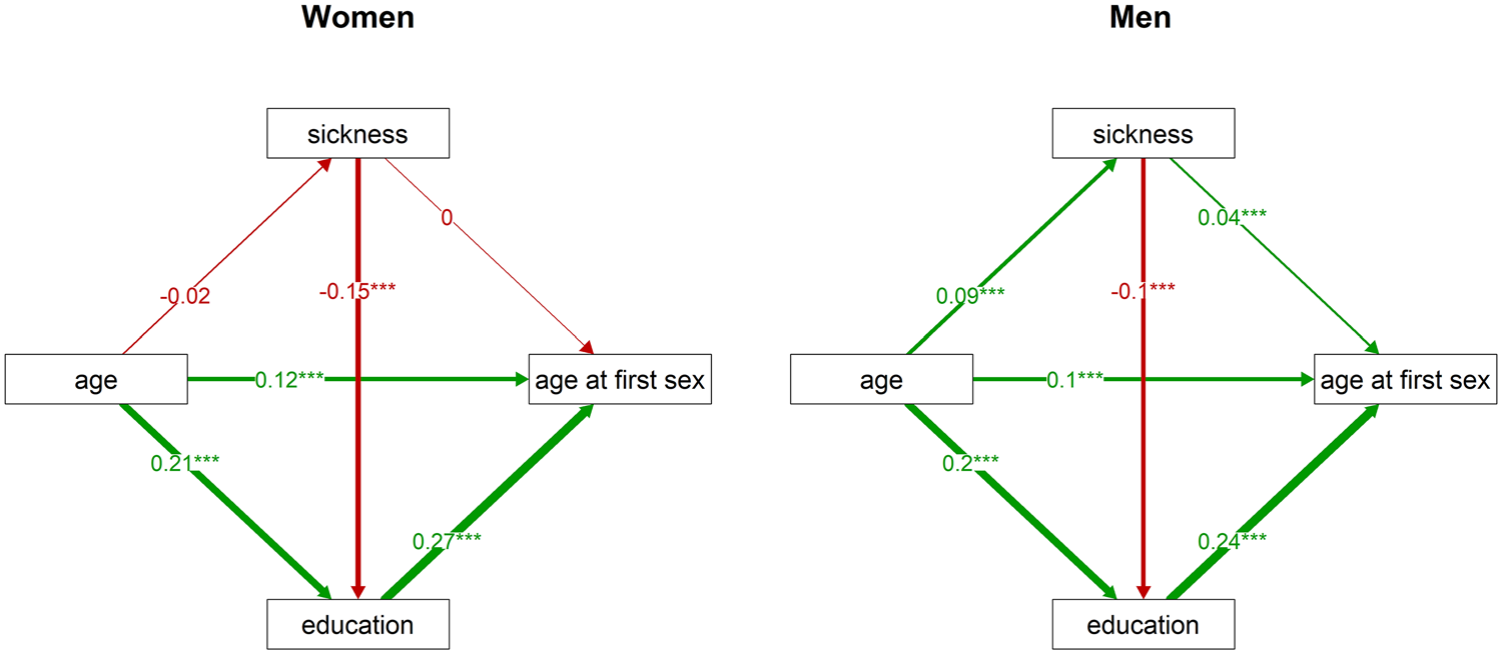
Direct and indirect effects of the sickness index on age at first sexual intercourse in women and men. This figure visualises the results of path analyses: it demonstrates relations between age at first sexual intercourse, the sickness index, education, and age, separately for women (left) and men (right). The numbers at arrows show standardised parameter estimates. Associations with p values under 0.001 are marked by ‘***’.

To test this hypothesis, we studied the relation of the three variables using a path analysis. In women, the sickness index had no direct effect on age at first sexual intercourse (Fig. 2). It did, however, predict education and education had a direct effect on age at first sexual intercourse, which resulted in an indirect negative effect of worse health on age at first sexual intercourse. We performed the same path analysis for men. Here, we observed a direct positive effect of the sickness index on age at first sexual intercourse. The indirect (education-mediated) effect of the sickness index was negative, as it was in women, but weaker than the direct effect of the sickness index. As a result, the overall impact of impaired health on age at first sexual intercourse in men was positive, i.e. less healthy men start their sexual life later. Results of the path analyses thus support our hypothesis regarding the mediating role of education in the association between impaired health and earlier initiation of sexual life only in women.

**Figure 3:**
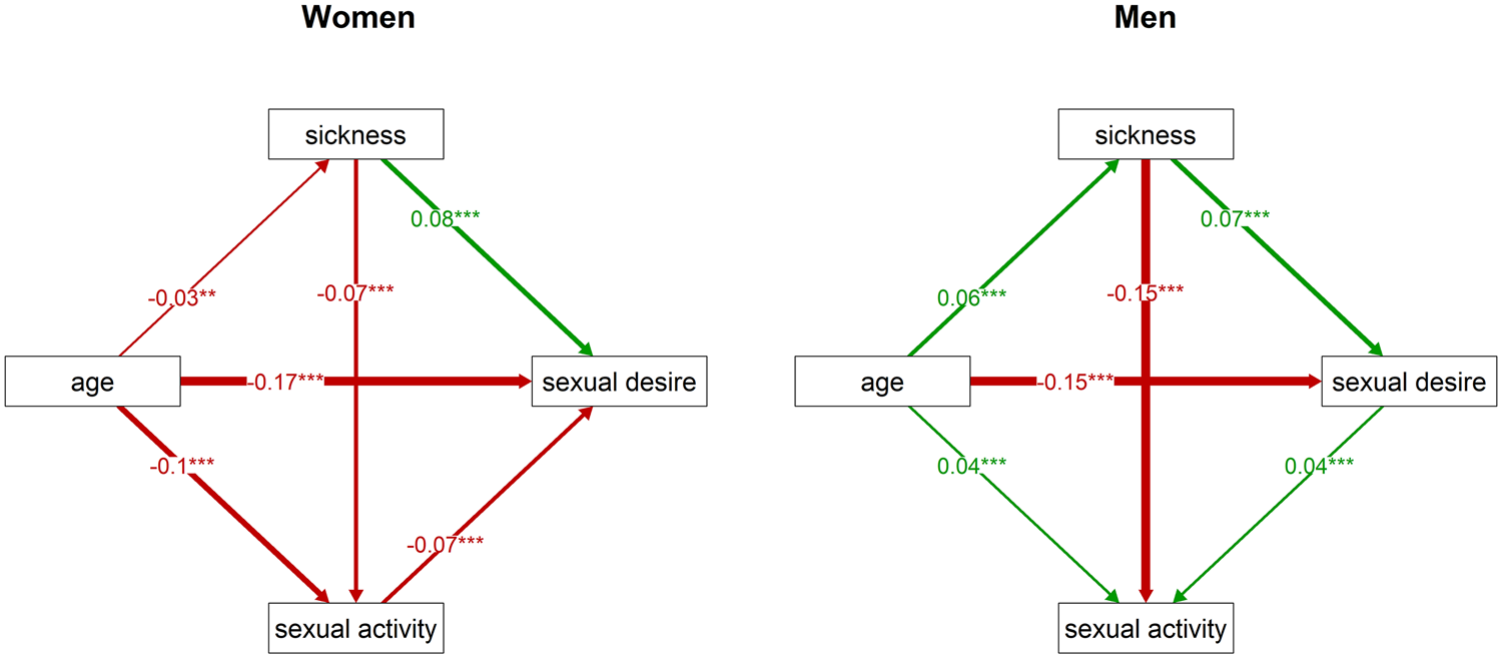
Direct and indirect effects of the sickness index on sexual desire in women and men. This figure charts the results of path analyses: it shows relations between sexual desire, the sickness index, sexual activity, and age, separately for women (left) and men (right). The numbers at arrows show standardised parameter estimates. It is more parsimonious to expect that in men, sexual desire positively influences sexual activity than that sexual activity positively influences sexual desire. The direction of the arrow between sexual desire and sexual activity was therefore reversed in the men’s model (see “Discussion”). Associations with p values under 0.01 are marked by ‘**’, and those with p values under 0.001 are marked by ‘***’.

Further, GLM analyses revealed a significant positive association between the sickness index and sexual desire, and a negative association between the sickness index and sexual activity in both sexes (Table 2). A simple explanation of these relations could be that higher sexual desire of people with impaired health is a direct consequence of their lower sexual activity, i.e. their involuntary sexual abstinence. Again, we tested this hypothesis using a path analysis, which showed that the sickness index has a direct positive effect on sexual desire in both sexes even when sexual activity was included in the model (Fig. 3). Moreover, in men, the association between sexual activity and sexual desire was positive instead of negative. This finding is incompatible with the suggested explanation of higher sexual desire mediated by lower sexual activity in subjects with impaired health.

**Figure 4:**
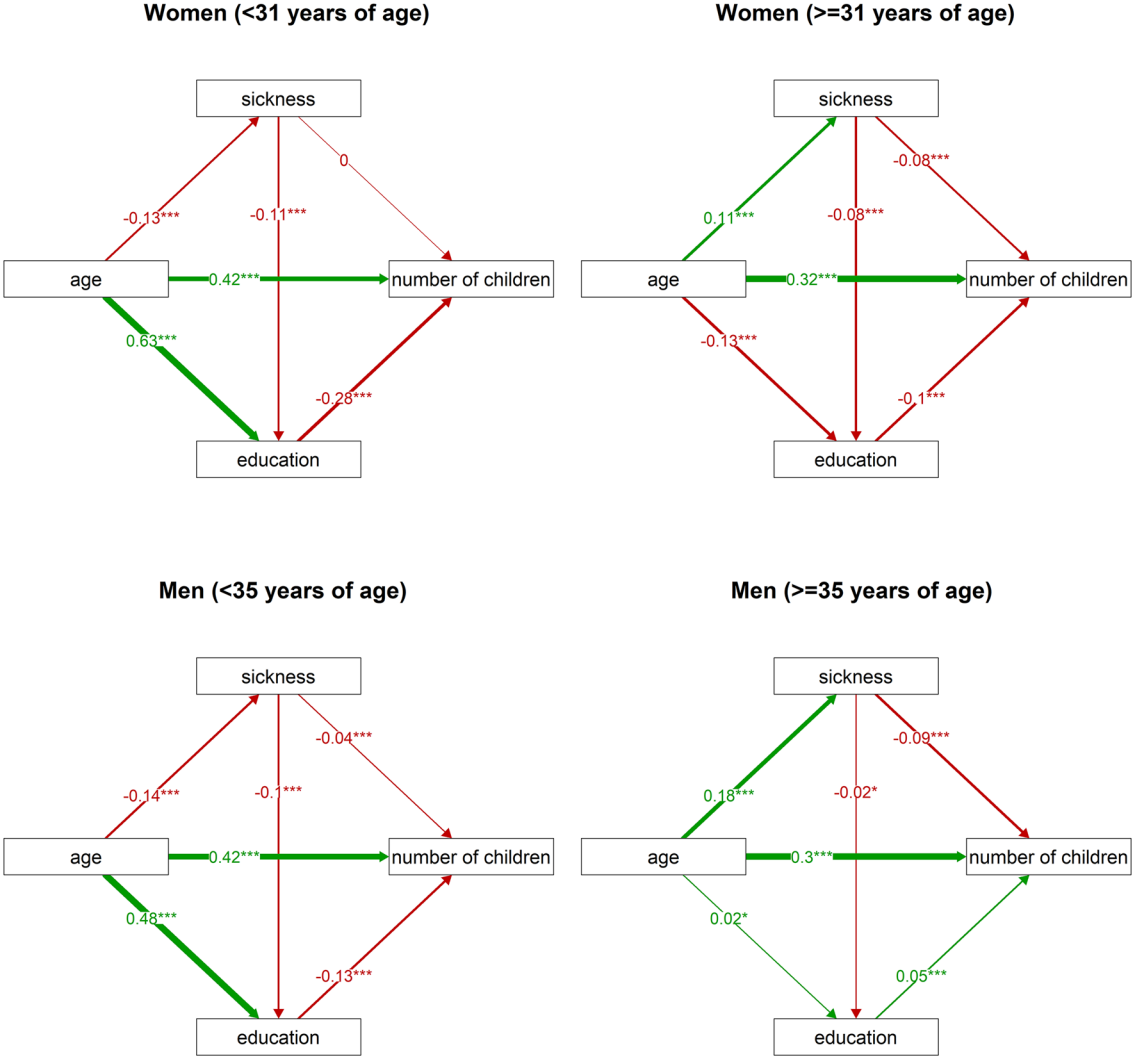
Direct and indirect effects of the sickness index on the number of children in women and men. This figure shows the results of path analyses: it visualises relations between the number of children, the sickness index, education, and age, separately for women under 31 years of age (top left), women over 31 years of age (top right), men under 35 years of age (bottom left), and men over 35 years of age (bottom right). The numbers at arrows show standardised parameter estimates. Associations with p values under 0.05 are marked by ‘*’, those with p values under 0.01 are marked by ‘**’, and those with p values under 0.001 are marked by ‘***’.

We have also explored the mediating role of education in the association between the sickness index and the number of children given the negative correlation between the sickness index and education. We expected that impaired health would have a positive (education-mediated) effect on the reproduction rate, thus leading to a higher number of children at a younger age, and a negative effect on the reproduction rate at older age. To this end, we analysed data for women and men under the median age (younger women/men) and over this median (older women/men) separately. As predicted, the sickness index had an indirect (education-mediated) positive effect on the number of children in women and men under the age of 31 and 35, respectively (Fig. 4). In men under the age of 35, however, the sickness index had also a much stronger direct negative effect on the number of children. In women aged over 31, the indirect positive effect of the sickness index was weaker, and a new, much stronger, direct negative effect of the sickness index on the number of children had emerged (Fig. 4, top right). In men over the age of 35, the indirect effect of the sickness index on the number of children turned from positive to negative because the effect of education on the number of children changed from negative to positive (Fig. 4, bottom right). The direct negative effect of the sickness index on the number of children was twice stronger in the subpopulation of older men than in the subpopulation of younger men.

Education level as well as behaviours related to sexual life and reproduction could be either the cause or the effect of an individual’s health. To tackle the problem of causality, we examined the effect of Rh phenotype on all variables related to sexual life and reproduction which were tested already in connection with health. It has been reported that Rh-positive subjects enjoy statistically better health than Rh-negative individuals^48–50^. At the same time, the Rh phenotype cannot be affected by environmental factors including education and reproduction-related behaviour.

**Figure 5:**
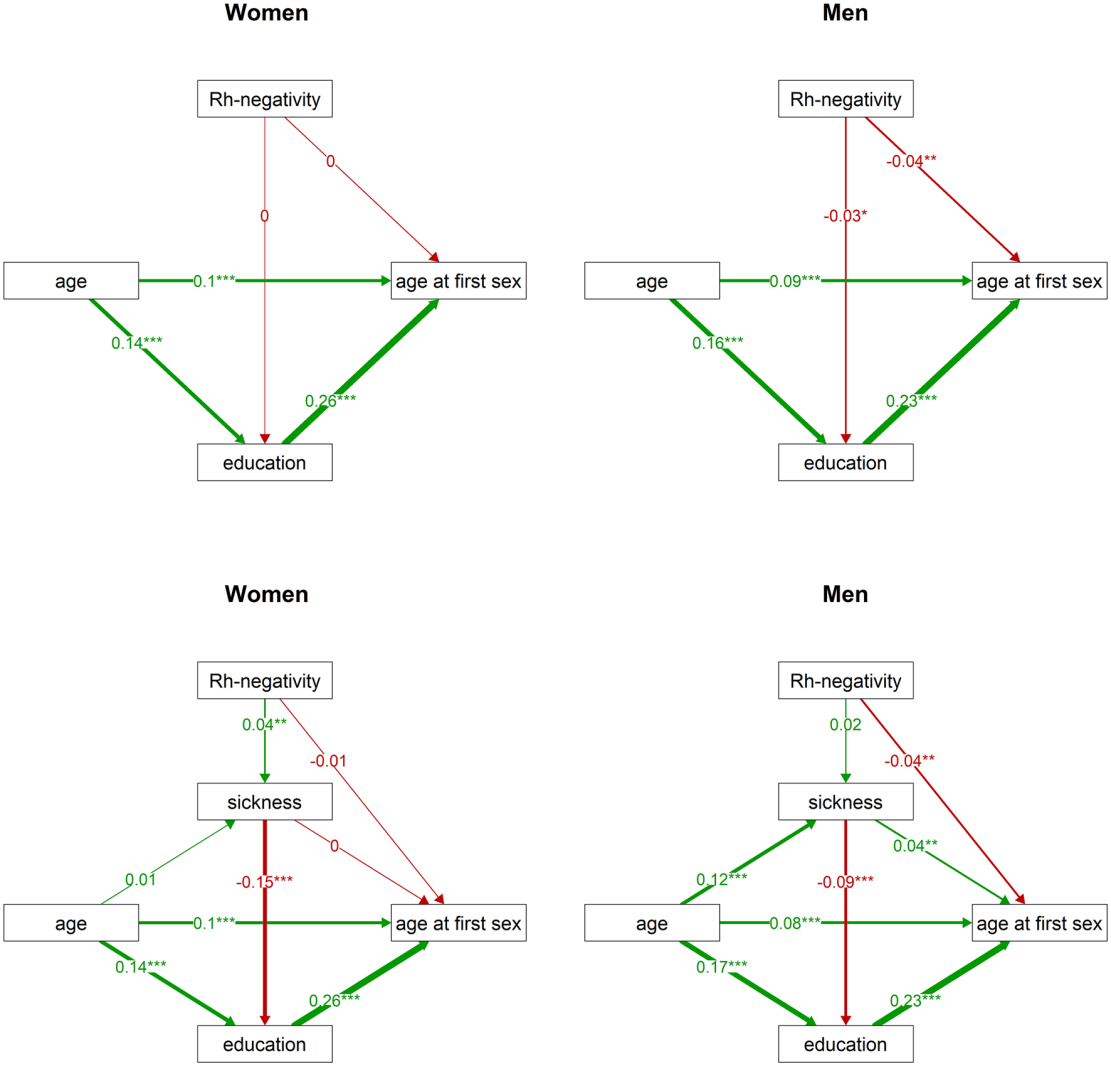
Direct and indirect effects of Rh-negativity on age at first sexual intercourse in women and men. This figure displays the results of path analyses of relations between age at first sexual intercourse, Rh-negativity, education, and age (top), and relations between these variables and the sickness index (bottom) for women (left) and men (right). The numbers at arrows show standardised parameter estimates. Associations with p values under 0.01 are marked by ‘**’, and those with p values under 0.001 are marked by ‘***’.

GLM analyses (see Table 3) revealed that in men, Rh-negativity indeed has a negative effect on age at first sexual intercourse and a positive effect on the number of sexual partners. In women, path analysis that included only the Rh phenotype showed no association between Rh-negativity and age at first sexual intercourse (Fig. 5, top left). On the other hand, once both Rh phenotype and the sickness index were included, path analysis showed that in women, Rh-negativity leads to worse health (Fig. 5, bottom left). It seems therefore that in women, Rh-negativity indirectly predicts lower age at first sexual intercourse. In men, the positive effect of Rh-negativity on the sickness index was not significant (Fig. 5, bottom right—standardised parameter estimates 0.02 in men vs. 0.04 in women). In men, however, the analysis revealed a direct negative effect of Rh-negativity on age at first sexual intercourse. Also in men, GLM analyses showed a significant effect of interaction between the Rh phenotype and age on sexual desire (Table 3). Visual examination of the corresponding variables showed that sexual desire was higher in older Rh-negative men (over the age of 30) than in older Rh-positive men (Supplementary Fig. S4).

We have also explored the mediating role of education in the association between the Rh phenotype, the sickness index, and the number of children using a path analysis. As in the case of path analyses for the effects of the sickness index, we analysed sets of women and men under and over the median age separately. A positive effect of Rh-negativity on the sickness index was significant in women and men under the age of 31 and 35, respectively (Supplementary Fig. S5, left, women: p = 0.001; men: p = 0.044).

## Discussion

The results of our preregistered tests show that less healthy people display signs of a faster life strategy, such as lower age at menarche in women and higher levels of sexual desire and earlier reproduction in both women and men. A subsequent post hoc test in the exploratory part of the study also supports the hypothesis about an earlier initiation of sexual life in women. Some of the effects of worse health are probably direct, others mediated by lower investment in education. Several results went the opposite direction than we hypothesised in the preregistration, but those results showed a clear pattern because they all concerned the negative effect of impaired health on sexual behaviour. In essence, it turned out that less healthy people tend to exhibit lower sexual activity and have fewer sexual partners and less healthy men also start their sexual life later. With the exception of this negative effect of worse health on sexual activity and promiscuity (discussed below), all other results are consistent with the hypothesis that in humans, impaired health serves as a signal for switching from a slower to a faster life strategy.

With some exceptions, which are discussed below, our results concur with previously published observations. Earlier studies reported that lower age at menarche is associated with certain chronic diseases such as cardiovascular disease, asthma, diabetes, or cancer^55–61^. Earlier menarche was also observed in girls who had a lower birth weight or were thinner at birth^62–64^. Similar to worse health, low birth weight has been associated with an increased risk of mortality^65, 66^. It is possible that intrauterine stress resulting in lower birth weight reduces life expectancy and consequently leads to a shift to earlier sexual maturation.

Our results may seem to contradict the conclusions of a review article by Gluckman and Hanson^67^ which described a relation between impaired children’s health and delayed menarche. It should be noted, however, that the association discussed in the abovementioned article is rather the result of childhood malnutrition in developing countries than a direct impact of worse health per se^68–71^. Gluckman and Hanson^67^ had also argued that postnatal nutritional deprivation delays menarche as it were in expectation that the environment and nutritional situation might improve. After all, alternation between periods of adverse and favourable conditions of food availability can be regular, sometimes even cyclical, and therefore predictable. In a situation of food scarcity, it is thus adaptive to postpone reproduction to a period of food availability^1^. In contrast, individuals who are in worse health have lower certainty of future improvement of their health status because in most cases, current bad health indicates even worse health in the future^72^. Unlike poor nutritional status, impaired health should thus be associated with lower age at the onset of menarche.

Our results showed that sexual desire is negatively associated with better health. An examination of the significant association of sexual desire with the interaction between age and the sickness index revealed that sexual desire is higher in less healthy women when they are under 50 years of age (supporting the faster life strategy hypothesis) and in less healthy men when they are over 50 years of age. Higher sexual desire in older, rather than younger, less healthy men seems to contradict the faster life strategy hypothesis given the expected lower investment in future reproduction in less healthy individuals. Nevertheless, the results of a path analysis showed that in men, but not in women, the age has a positive effect on worse health even in our relatively young/middle-aged population sample. This association can amplify the effect of impaired health on sexual desire in older men.

A path analysis also showed that higher sexual desire in less healthy people is not the result of their lower sexual activity, because the effect of impaired health on sexual desire, although relatively weak, was direct. Moreover, in men, sexual desire correlated positively with sexual activity, which likewise contradicts the proposed explanation of the mediating role of lower sexual activity. It is highly likely that the relation between sexual activity and sexual desire has two opposing components. In essence, higher sexual desire usually results in higher sexual activity and sexual activity temporarily reduces sexual desire. The relative strength of these two opposing processes controls the resultant direction and strength of the activity–desire association. In a relatively young population, such as the sample in this study, it is men, rather than women, who tend to initiate sexual activities^73^. This is why in men, the positive effect of sexual desire on sexual activity outweighs the negative effect of sexual activity on sexual desire. The opposite might be true of women where, additionally, their sexual activity may be affected by both their own sexual desire and sexual desire of their male partners.

The correlation between impaired health and age at first sexual intercourse was negative and nonsignificant in women but positive and significant in men. Nevertheless, a path analysis revealed a weak indirect negative effect of worse health on age at first sexual intercourse mediated by education in both sexes. Impaired health is likely to lead to lower investment in the future, which manifests itself by lower investment in education. It has been shown that a decision not to acquire higher educational attainment leads to earlier initiation of sexual life^74, 75^ (but see a discussion of possible opposite causality below). We suggest that this decision may be part of the faster life strategy in individuals in worse health. Our results showed in men, this indirect effect of worse health on age at first sexual intercourse was outweighed by a direct positive effect of worse health (later initiation of sexual life in individuals with impaired health) since it was almost twice as strong as the indirect effect. It is thus possible that the adverse effects of worse health on viability and ability to have sex start affecting men earlier than women, even during their young adulthood.

We found no evidence of a positive effect of worse health on sexual activity or the number of sexual partners. In contrast to our expectations, but in agreement with already published results^51^, our results show that less healthy people are less sexually active and less promiscuous. It could be argued that in modern society, rationality plays an important role and prevents the expected increase in sexual activity. The urge to higher sexual activity or promiscuity, i.e. higher sexual desire, can be suppressed by a rational decision not to seek sexual partners when knowing about one’s own impaired health. Of course, another, altogether more prosaic, explanation of lower sexual activity and lower number of sexual partners in less healthy individuals is that impaired health interferes with their wellbeing and various physiological functions. This may be why less healthy people engage in less sexual activity, including recreational sexual activity, despite their higher sexual desire. Moreover, people perceived as less healthy are rated as less attractive^76, 77^ and may thus be less preferred as sexual partners, which would contribute to this association. These related explanations are supported by the observed distribution of sexual activity and the number of sexual partners in specific age and health strata. In our study, the decline of both sexual activity and promiscuity with age was more rapid in less healthy individuals (Supplementary Figs. S2–3).

Our analyses also revealed that individuals with impaired health have fewer children in total. This finding contrasts with the results of Chua et al.^26^ who found a positive effect of worse health on the number of children. Our results are, however, in agreement with recent findings of Woodley of Menie et al.^78^ which show a positive association between slower life strategy and the number of children. They proposed that the lower fecundity of individuals with a faster life strategy could be the result of their stricter contraceptive usage in our day and age because conscious family planning is not a part of their biologically programmed strategy.

When registering the hypotheses concerning initiation of reproduction, we expected that less healthy people would have more children at a younger age and fewer children at an older age than healthier people. We observed this effect in our contour plots (Fig. 1). This observation is in accordance with our preregistered hypotheses as well as with the results of Waynforth^38^, who too demonstrated earlier reproduction in people with impaired health. In our study, path analyses showed an indirect (education-mediated) positive effect of worse health on the number of children in both younger women and men. In the subpopulation of younger men, worse health also had a direct negative effect on the number of children and this effect was much stronger than the indirect (education-mediated) effect. Overall, therefore, less healthy younger men had fewer children. It seems that impaired health directly manifests itself as problems with sexual activity, fertility, and in men probably also potency. As mentioned above, these adverse effects of worse health possibly start affecting men earlier than women.

In older individuals of both sexes, we also found a direct negative effect of worse health on the number of children. This negative effect of worse health was about twice stronger in older men than in younger men. In older women, this negative effect of worse health also multiple times outweighed the indirect (education-mediated) positive effect of worse health on the number of children. It seems that as health deteriorates with aging, the importance of the direct negative impact of worse health on fecundity grows. The faster life strategy ceases to bring positive effects on biological fitness later in life. It is likely that the faster life strategy adopted by less healthy people, which exhausts the organism at a younger age, has itself some negative long-term consequences on reproduction capacity at an older age, such as general fatigue and even greater health impairment^32, 33, 79–83^. Conversely, the slower life strategy of healthier people becomes more advantageous with increasing age. In people with a slower life strategy, higher investment in maintenance rather than reproduction early in life results in their higher reproductive potential at an older age and ultimately a higher lifetime fecundity.

In older men, worse health had not only a direct but also an indirect negative effect on the number of children, because the level of educational attainment was positively associated with the number of children. We assume that in older men, higher education implies a higher social status and better economic situation. This gives men, though not women, an important advantage on the mating market and it ultimately leads to their higher fecundity. The resulting disadvantage of less educated and less healthy men only slightly contributes to the direct negative effect of impaired health on the number of children at an older age.

Before the advent of modern medicine, the direct negative effects of worse health on fecundity later in life were probably much stronger than today. Improvements in medical care and improved accessibility of healthcare not only allow for a longer lifespan but also offer the possibility of reproduction to less healthy people with fertility problems. Currently, healthier individuals have on average slightly more children than less healthy people, but if the current trend of decline in human fecundity and improvements in medical care were to continue, the long-term consequences of the interplay between the direct and indirect effects of worse health could be paradoxical and disturbing. In essence, less healthy women and men would end up having higher lifetime fecundity than healthier individuals. A shift towards a faster life strategy with attendant higher fecundity at a young age in connection with thanks to modern medicine merely negligible decrease in the reproduction rate at an older age could result in an increase in the frequency of genes predisposing to various diseases in the gene pool of modern societies. This effect would be even more pronounced if the faster life strategy resulting in higher sexual desire also led to higher levels of sexual activity. It can also be expected that the positive effect of worse health on fecundity at a young age would be much stronger in countries where modern contraception is not as easily available as, for instance, in the Czech Republic and in countries or within social groups where birth control is forbidden or frowned upon, for instance for religious reasons.

As expected, and in agreement with the results of previously published studies^48–50^, Rh-negative women and Rh-negative younger men were significantly less healthy than Rh-positive subjects. In women, Rh-negativity therefore indirectly, via impaired health, resulted in lower age at first sexual intercourse, while Rh-negative men had likewise lower age at first sexual intercourse but also a higher number of sexual partners. On the other hand, our results support the hypothesis regarding earlier reproduction neither in Rh-negative people, nor in Rh-negative less healthy people, although we found that Rh-negative men ultimately do have more children (beta coefficient = 0.046, p = 0.033, one-tailed test).

At face value, the results of analyses of effects of Rh-negativity on age at first sexual intercourse and the number of sexual partners in men seem to contradict the results of analyses of effects of worse health on the same variables (see Tables 2 and 3). The effect of Rh-negativity but not the effect of impaired health was in accordance with our preregistered hypotheses. We assume that both Rh-negativity and worse health might act as independent signals for switching to the faster life strategy. Rh-negativity, or more probably physiological parameters associated with Rh-negativity, can be used by a developing organism, even at prenatal stage, as a predictor of a higher likelihood of future poor health. The onset of many disorders, however, depends on a combination of genetic and environmental factors, such as encounters with various pathogens. It has been, for instance, shown that certain negative effects of Rh-negativity can be observed only in subjects infected with the *Toxoplasma*^43, 44, 47, 84^. Rh-negativity also seems to make individuals more sensitive to bacterial infections, aging, and smoking^48, 49^. This is why some predisposed individuals, i.e. Rh-negatives, may avoid poor health. This explanation is supported by the significance of a three-way Rh–sickness–age interaction (Supplementary Table S4), which shows that in men, the effect of Rh-negativity on sexual desire depends on whether they have impaired health or not. Rh-negative people may switch to a faster life strategy but if they are healthy, they escape the negative impacts of their predisposition and suffer no impairment to physiological functioning and general wellbeing. As a result, healthy Rh-negative men may indeed start their sexual life earlier and have more sexual partners and/or more children than men with impaired health who are prevented by diseases from expressing some of the signs of a faster life strategy.

### Strengths and limitations of the study

So far, only a handful of studies examined the direct impact of health on the adoption of slow or fast life strategy in humans^36–38^. Moreover, no study as yet tried to determine whether individual health affects the age of sexual maturation and behaviours related to sexual life aside from just the timing of reproduction. The first strength of the present study is the inclusion of a hypothesis about earlier sexual maturation (age at the onset of menarche) in less healthy women among the studied relations. Unlike the other reproduction-related variables examined in this study and elsewhere, age at menarche is a genuinely biological factor because its timing cannot be influenced by a conscious decision. Moreover, the onset of menarche takes place prior to the beginning of higher education, which implies that education cannot play a mediating role. These factors make the age at menarche a perfect variable for investigating life history strategies in humans. The second strength of this study is that we included into our analyses the effects of the Rh phenotype on variables related to life strategies. Rh genotype cannot be influenced by environmental factors, which eliminates potentials problems with the direction of causality in the observed relations. The third and fourth strengths of the present study consist in the large number of participants (32,911) and the fact that the study was promoted in most general terms (‘the study tests certain hypotheses of evolutionary psychology and parasitology’). The specific subject of the study was not revealed to the participants and therefore could not influence the process of their self-selection. The fifth strength of the study was its preregistration, which eliminated the risk of artefacts related to data dredging, cherry-picking, or p-value fishing.

The main limitation of this study is that the independent variable sickness index was computed from responses to questions regarding participants’ current health. For optimal testing of our hypotheses, we would have to have access to information about participants’ health in childhood and adolescence. Impaired health later in life can be the consequence of a genetic predisposition or suboptimal development or a result of a lifestyle related to faster life strategy itself (e.g. risky behaviour, unhealthy diet, early reproduction). Longitudinal studies had moreover shown that impairments of childhood and adolescent health are associated with poor health in middle age even after controlling for educational attainment and socioeconomic status^85, 86^. These findings suggest that current health might be an accurate proxy of health in early life when life strategies could be mostly calibrated. In any case, though, the observed effects should be verified in future using some objective health-related data, such as medical records from childhood.

In one of our preregistered hypotheses, we expected to find a difference in the number of children born to people in worse or better health at a younger and older age. The questionnaire, however, contained only questions about respondents’ age and number of children and not a question about respondents’ age at the time of birth of their offspring. Therefore, we did not know whether the children of older subjects were born when the respondents were young or older. It is of the utmost importance to ask not only about number of children but also about the age at the time of birth of offspring in future studies.

Similarly, we would like to compare total, i.e. lifetime, fecundity of healthier and less healthy individuals. For obvious reasons, we have only data from living, mostly young and middle-aged subjects who have not yet realised their full reproductive potential. In part, we addressed this issue by using distance-weighted least-squares extrapolation when drawing the XYZ contour plots. This extrapolation enabled us to compare the number of children in subjects of different health status even in the higher-age strata. This method, however, cannot eliminate the problem of difference between age cohorts. In the Czech Lands, the mean fecundity changed dramatically over the past century and these changes can distort the pattern of changes of actual fecundity in particular age strata.

In our analyses, we assumed that worse health was the cause of lower educational attainment, but the causality could also be the opposite: education can affect health. In fact, this relationship is probably bidirectional and it is difficult to assess which direction is more important^87^. Nevertheless, we know that years of education, grade point average, educational attainment, or qualification for a college are influenced by a number of health indicators, including lower birth weight^88, 89^, health-threatening exogenous events during prenatal development or in early childhood, such as exposure to an influenza pandemic^90^, radioactive radiation^91^, lead^92^, or parasitic infections^93^, as well as general health problems^85^, migraines^94^, or mental health difficulties^95, 96^ in childhood. Most importantly, we also detected an association between education and (gene-determined) Rh-phenotype, where only the effect of Rh-phenotype on education—and not the opposite direction—can come into play. We therefore assume that especially in young participants of our study, the effect of health on educational attainment may be stronger than the effect of educational attainment on health.

We gathered information about health and behaviours related to sexual life from respondents’ self-reports in an anonymous questionnaire survey. The prevalence of 24 mental health disorders^97^, as well as percentages of members of various churches^98^ obtained via our questionnaire are in reasonably good agreement with data gathered in representative national surveys, which suggests that data collected by this method are relatively reliable. Still, respondents could provide inaccurate information, especially about variables related to sexual life. Nonetheless, Monte Carlo models show that analyses of data containing stochastic noise can generate only false negative results, not false positive results^99^. Even so, we could have a problem with systematic bias. Some subpopulations of respondents, for instance those who score high on neuroticism or consciousness, hypochondriacs, or pathological liars, might provide inaccurate information about both their health and their behaviours related to sexual life and reproduction. It is urgently needed to repeat our study in future on a population for which some data based on medical records is available.

In the present study, we have detected many statistically significant associations between focal variables, but the sizes of these effects ranged mostly between 0.01 and 0.02. Formally, such effects fall into the category of small (but not negligible) effects. Cohen^100^ in several places of his seminal monograph remarks that such small effects are typical of many fields of biology and psychology, mostly because of large variance of biologically relevant variables measured in polymorphic outbreed populations. Paradoxically, the observed effects tend to be even smaller when measured in large population samples. Because many biological variables are heavy-tailed, rather than normally distributed, large samples are more likely to contain rare outliers, which might increase the total variance of focal variables. Similarly, smaller effect sizes can be expected when studying the effect of any factor on variables closely connected with biological fitness, such as variables related to reproduction and sexual behaviour. The most important reason for the small effect sizes obtained in this and similar studies is, however, that we do not measure the actual correlation between the focal traits (e.g. between sickness and sexual activity). What they measure is just a correlation between variables correlated with the focal traits, in our case, the willingness and ability to report various problems and the willingness and ability to report the monthly frequency of sexual intercourse over the past year. If the correlation between sickness and sexual activity was 0.5, correlation between sickness and our sickness index was 0.5, correlation between sexual activity and reported frequency of sexual intercourse was also 0.5, and no other relations came into play, we would obtain a correlation between the sickness index and reported frequency of sexual intercourse of just 0.125, which corresponds to effect size 0.016.

## Conclusion

Results of the present study show that worse health is associated with lower age at menarche in women, higher sexual desire, earlier reproduction, a lower number of children in both women and men, and indirectly also with earlier initiation of sexual life in women. We also observed signs of a higher investment in reproduction in men with Rh-negative phenotype who are (statistically) genetically predisposed to worse health. The observed associations suggest that impaired health and possibly even a genetic predisposition to it could play the role of a signal for switching to a faster life strategy. Faster life strategy induced by impaired health led to earlier reproduction but also lower lifetime fecundity, which shows that switching to a faster life strategy only partly compensates for the direct negative (physiological) impacts of impaired health on fecundity.

Anthropological data suggest that the advent of agriculture which led to a change of diet to a nutritionally imbalanced one and to settlement of populations, i.e. transition to a sedentary lifestyle and ultimately to life in much closer proximity to other humans, resulted in a dramatic worsening of health in general human population^101–105^. This worsening of health probably caused an automatic switch to a faster life strategy in average members of the human population. Taking this into account, we suggest that the improvement in living conditions and especially in general public health which took place over the past 150 years in developed countries^42^ enabled a return to the original slower life strategy of hunters and gatherers. Such fast-to-slow life strategy shift offers a new biological explanation for the well-known phenomenon of the demographic transition, that is, of the fecundity decline in rich developed countries.

## Materials and methods

### Subjects

The internet questionnaire was promoted and distributed as a ‘study that tests certain evolutionary psychological and parasitological hypotheses and contains many questions related to sexual life’ (for details see Ref.^106^). Participants were informed about the general aims of the study on the first page of the questionnaire. Neither health nor life strategies were explicitly mentioned in the aims of the study during the recruitment campaign. Only subjects who provided their informed consent (including a confirmation of being at least 15 years old) by pressing the corresponding button could participate in the study. The questionnaire was written in Czech and due to the highly ethnically homogeneous population of the Czech Republic, nearly 100% of participants were Czech or Slovak nationals. Between 22 January 2015 and 6 September 2017, we obtained data from a total of 48,032 respondents. The project, which consisted of several unrelated studies, as well as the method of obtaining electronic consent to participation in the studies, were approved by the institutional review board of the Faculty of Science, Charles University in 2015 (No. 2015/01). The present life history study was approved by the same IRB in 2018 (No. 2018/04). All methods were performed in accordance with the relevant guidelines and regulations of IRB of the Faculty of Science, Charles University.

### Questionnaire

The online survey consisted of several questionnaires aimed at collecting various socioeconomic, demographic, health-related, sexual life- and reproduction-related, psychological, behavioural, and epidemiologic data. The survey also contained the Sexual Preferences and Behaviours Inventory 2015 (SPBI-2015)^106^ and Revised Sociosexual Orientation Inventory (SOI-R)^107^. All in all, the survey consisted of about 700 questions and the mean time needed for completion was about 101 min (median 96 min).

In the present study, we used only responses to questions concerning health, Rh phenotype, sexual life, and reproduction. As outcome variables, we analysed the age at menarche, age at first sexual intercourse, and the number of children as direct responses to the corresponding questions. Sexual desire was computed as the sum of three items from the Revised Sociosexual Orientation Inventory, namely the frequency of having fantasies about having sex with someone the respondent is not in a committed romantic relationship with, the frequency of experiencing sexual arousal when the respondent is in contact with someone they are not in a committed romantic relationship with, and the frequency of having spontaneous fantasies about having sex with someone the respondent had just met (each item has had nine-point scale anchored with never—code 1, and at least once a day—code 9). We also estimated subjects’ sexual activity as the average number of sexual intercourse per month in the past year (an eight-point scale anchored with none—code 1, and over 30—code 8). The last outcome variable, the number of sexual partners, corresponded to the number of sexual partners in the past year (a nine-point scale anchored with 0—code 1, and 20 or more—code 9).

To obtain the main predictor for testing hypotheses h1–h6 and h8–h12 (see Table 1), we calculated a sickness index as the arithmetical mean of Z-scores of 11 health-related variables. The sickness index was measured based on responses to five health-related ordinal variables (0–8 scale), in particular the number of antibiotic treatments a respondent used in the past year, the number of different kinds of drugs prescribed by a medical doctor which a respondent was using daily, the number of different kinds of non-prescription drugs or food supplements a respondent used on a daily basis, the number of visits to a general practitioner in the past year, and the number of different medical specialists a respondent visited in the past year. The sickness index also included six health-related semi-continuous variables (0–100 scale), namely, intensity of physical health problems, intensity of mental health problems, intensity of suffering with anxieties, depressions, manias, and obsessions. The sickness index was calculated from variables used for the computation of indices of health problems in several previously published studies^51, 97^. In contrast to them, we computed only one index of sickness instead of two separate indices reflecting physical and mental health. The original variables concerning current health status were selected by a general practitioner^49^.

The binary variable Rh-negativity was used as a predictor for testing hypotheses h7 and h13. We also examined potentially confounding variables including sex, age, socioeconomic status (0–100 scale), current partnership (binary variable), height, size of place of residence where the respondent spent most of the childhood (six categories: < 1000 inhabitants, 1000–5000, 5000–50,000, 50,000–100,000, 100,000–500,000, Czech or Slovak capital), highest education level achieved (the original eight categories transformed to five categories: primary school or secondary vocational school without A-level leaving certificate, complete secondary or higher education with A-level leaving certificate, bachelor’s, master’s, doctoral), and church membership (binary variable).

### Statistical analysis

Prior to statistical analyses, we filtered out records from less than 2% of subjects who provided a suspicious combination of body height, weight, and age, and/or reported having Alzheimer’s or Parkinson’s disease, more than nine mental health disorders, an unrealistically high number of children, or unrealistically low age at first sexual intercourse. We also deleted all records of subjects who did not follow the instructions and tried to participate in the study despite being less than 15 years old. For different analyses, we used in our study two sets of the same data^108^ which differed in the unsubstituted variables involved. The first set contained data from 29,827 subjects who provided information at least about their sex, age, current partnership, and church membership. In accordance with the preregistered protocol, we substituted all the missing data in this set (except age and binary variables) with population means. We used this dataset merely for analysis with multivariate multiple GLM. The second set included all records containing information at least about sex, age, and some information about respondents’ health. In this dataset, we substituted with population means only the potentially confounding variables (socioeconomic status, height, size of place of residence, and education). Outcome variables and predictors remained unaffected. This set contained data from 32,911 subjects and was used for testing all thirteen hypotheses in the confirmatory part of the study as well as for follow-up exploratory analyses. The number of subjects varied in each dataset and model depending on the variables included (the second dataset was larger than the first because one model tested on it included no binary variables which would reduce the number of complete records).

The distribution of all dependent (semi-continuous and ordinal) variables in both datasets was visually checked and categories of ordinal variables containing less than 3% of subjects were merged with adjacent categories. The variables sexual desire in women and number of sexual partners had highly asymmetric distributions, which is why we transformed these variables by logarithmisation to achieve semi-normal distributions. In both datasets, we computed new variables: sickness index as the arithmetical mean of Z-scores of 11 health-related variables (see section “Questionnaire” for details), and sexual desire as the sum of three items of the Revised Sociosexual Orientation Inventory, separately for women and men. All variables (except for the binary ones) were standardised prior to analyses by computing Z-scores. Following the preregistered protocol, we computed correlations between all output variables and potential covariates using a nonparametric partial Kendall correlation test with age as a covariate. Potential covariates with partial Kendall’s Tau > 0.02 or < − 0.02 were included in subsequent statistical models.

All thirteen hypotheses were analysed with general linear regression models (GLM). We performed a multivariate multiple GLM analysis with all sexual life- and reproduction-related variables as dependent variables and age, all potential covariates with partial Kendall’s Tau > 0.02 or < − 0.02, the sickness index, and interaction between the sickness index and age as independent variables. Each of the thirteen specific hypotheses was analysed with subsequent univariate GLM analysis. For analysis with GLM, we used the Type III sums of squares since in this type, the order of variables in a model does not matter. Nevertheless, GLM analyses with Type I sums of squares provided almost the same results (Supplementary Table S5). In the exploratory part of the study, we conducted a path analysis to test the role of potentially mediating variables. To address the issue of multiple tests artifact, we used the Benjamini–Hochberg correction with false discovery rate set as 0.1 for the set of preregistered tests. Statistical analysis was performed with statistical software IBM SPSS v. 21 (GLM, Kendall correlation tests, Cox regression, descriptive statistics), Statistica v. 10.0. (XYZ contour plots), and R v. 3.5.2^109^ using the ‘lavaan’^110^ and ‘semPlot’ package^111^ (path analysis). To compute partial Kendall’s Tau and the significance of each variable after controlling for age, we used an Excel spreadsheet available at (https://doi.org/10.6084/m9.figshare.12102810.v1).

### Differences between the preregistered and actually implemented protocol

1. In the preregistered protocol, we planned to substitute all missing data (except for the Rh phenotype) with population means. It turned out, however, that after this substitution the distribution of all outcome variables was highly asymmetric with an artificial peak at the point of the population mean. This was clearly caused by the substitution of missing data by the mean. We have therefore decided to use the dataset with mean substitutions for all missing data only for analysis with the multivariate multiple GLM. We performed all other analyses with a dataset where only the missing data for potentially confounding variables were substituted by the population mean. It ought to be mentioned, however, that confirmatory analyses with complete data provided almost identical results (Supplementary Table S6).
2. While in the preregistered protocol, we opted for exclusion of records with a suspicious combination of over 14 mental health disorders, after a visual inspection of histograms we filtered out subjects reporting over nine mental health disorders.
3. In the present study, we standardised all variables (except for the binary ones) prior to analyses, which allowed us to present comparable coefficients of variables that originally had different scales. This operation was not mentioned in the preregistered protocol.
4. We had preregistered the use of one-tailed tests for testing our one-sided hypotheses but the tables and figures in this paper display the results of more stringent two-tailed tests. We took this step because the use of one-tailed tests was not preregistered for some associations presented in the same tables and figures. Ultimately, however, the results of one-tailed tests for the preregistered hypotheses were qualitatively identical to the corresponding results of more stringent two-tailed tests.
5. By mistake, we preregistered slightly different versions of hypotheses h7 and h13 than appear both in our approved protocol and are presented in Table 1. The preregistered hypotheses were as follows: ‘Rh-negative less healthy women/men have more children earlier in life and fewer children later in life than Rh-positive less healthy women/men.’ We have therefore tested both variants of the hypotheses and the results were the same: the data supported neither variant (see “Results”).

### Ethics

The research has been approved by the Institutional review board of the Faculty of Science, Charles University, No. 2018/04.

### Preregistration

The study was preregistered as Sýkorová, K., September 2017: ‘Relation between a health condition and life-history traits in humans’ on the website of the Open Science Framework (https://osf.io/wfbkr/).

## Supplementary Information

Supplementary Information.

## Data Availability

Dataset supporting this article can be accessed on Figshare (https://doi.org/10.6084/m9.figshare.12100623.v1).
