# Supplementary material for: Faster life history strategy manifests itself by lower age at menarche, higher sexual desire, and earlier reproduction in people with worse health
Source: Sci Rep. 2021 May 27;11:11254. doi: 10.1038/s41598-021-90579-8 (PMC8159921; doi:10.1038/s41598-021-90579-8)
Supplement: Supplementary file 1 — Supplementary Information. [file 41598_2021_90579_MOESM1_ESM.pdf]

Faster life history strategy manifests itself by lower age at menarche, higher sexual desire, and earlier reproduction in people with worse health

Kateřina Sýkorová [a,*] and Jaroslav Flegr [a,b]

[a] Department of Philosophy and History of Science, Faculty of Science, Charles University, Vinicna 7, 128 00, Prague, Czech Republic

[b] Department of Applied Neurosciences and Brain Imagination, National Institute of Mental Health, Topolova 748, Klecany, Czech Republic

[*]Corresponding author at: Department of Philosophy and History of Sciences, Faculty of Science, Charles University, Vinicna 7, 128 00, Prague, CZ
E-mail address: sykorok6@natur.cuni.cz

Supplementary materials

	question scale	women		men	
		mean	s.d.	mean	s.d.
age at menarche	direct responses	12.75	1.38		
age at first sexual intercourse	direct responses	17.33	2.54	18.73	3.35
sexual desire	scale 3-27	10.19	5.31	13.84	5.73
sexual activity	scale 1-8	4.26	1.88	4.05	1.86
number of sexual partners	scale 1-9	2.57	1.39	2.55	1.47
number of children	direct responses	0.91	1.08	0.94	1.15
number of antibiotics	scale 0-8	0.78	1.21	0.55	0.92
number of prescribed drugs	scale 0-8	0.48	1.00	0.53	1.16
number of non-prescription drugs	scale 0-8	0.86	1.17	0.60	1.04
number of visits to a general practitioner	scale 0-8	1.70	1.86	1.44	1.63
number of medical specialists	scale 0-8	2.69	2.12	1.95	1.76
physical health problems	scale 0-100	38.85	20.98	35.17	20.56
mental health problems	scale 0-100	31.93	22.40	26.03	20.31
anxieties	scale 0-100	22.48	26.23	13.90	20.19
depressions	scale 0-100	22.37	26.14	15.87	21.56
manias	scale 0-100	8.37	16.95	7.16	15.40
obsessions	scale 0-100	10.36	19.06	8.67	16.65
age	direct responses	31.55	12.02	35.81	12.42
socioeconomic status	scale 0-100	59.41	22.15	64.08	22.00
height	direct responses	167.82	6.49	181.35	7.12
size of place of residence	scale 1-6	3.36	1.72	3.48	1.67
education	scale 1-5	2.55	1.05	2.70	1.14
sickness index		0.00	0.62	0.00	0.59
	question code	N	%	N	%
current partnership	0	4,606	28.3	4,449	27.3
	1	11,656	71.7	11,828	72.7
church membership	0	11,792	87	14,220	87.4
	1	1,760	13	2,057	12.6
Rh-negativity	0	7,579	75.4	5,864	75.6
	1	2,479	24.6	1,888	24.4

Supplementary table S1. Descriptive statistics of all variables used in the study. This table shows the means and standard deviations (s.d.) of all semi-continuous and ordinal variables and numbers (N) and percentages (%) of all binary variables for women and men. For binary variables, code 0 indicates no current partnership, no church membership, and having a positive Rh phenotype; code 1 indicates being in a partnership, church membership, and having a negative Rh phenotype.

		number of antibiotics	number of prescribed drugs	number of non-prescription drugs	number of visits to a general practitioner	number of medical specialists	physical health problems	mental health problems	anxieties	depressions	manias	obsessions
number of antibiotics	♀	1.000										
	♂	1.000										
number of prescribed drugs	♀	**0.104***	1.000									
	♂	**0.080***	1.000									
number of non-prescription drugs	♀	**0.066***	**0.119***	1.000								
	♂	**0.058***	**0.119***	1.000								
number of visits to a general practitioner	♀	**0.416***	**0.201***	**0.084***	1.000							
	♂	**0.364***	**0.305***	**0.104***	1.000							
number of medical specialists	♀	**0.178***	**0.254***	**0.122***	**0.254***	1.000						
	♂	**0.168***	**0.255***	**0.128***	**0.274***	1.000						
physical health problems	♀	**0.065***	**0.109***	-0.001	**0.087***	**0.075***	1.000					
	♂	**0.061***	**0.135***	-0.001	**0.106***	**0.062***	1.000					
mental health problems	♀	**0.070***	**0.094***	**0.045***	**0.084***	**0.092***	**0.260***	1.000				
	♂	**0.042***	**0.066***	**0.062***	**0.066***	**0.087***	**0.257***	1.000				
anxieties	♀	**0.076***	**0.126***	**0.067***	**0.101***	**0.129***	**0.124***	**0.385***	1.000			
	♂	**0.063***	**0.074***	**0.096***	**0.078***	**0.123***	**0.108***	**0.379***	1.000			
depressions	♀	**0.077***	**0.138***	**0.057***	**0.094***	**0.114***	**0.124***	**0.416***	**0.578***	1.000		
	♂	**0.049***	**0.069***	**0.080***	**0.068***	**0.103***	**0.109***	**0.398***	**0.592***	1.000		
manias	♀	**0.060***	**0.033***	**0.046***	**0.063***	**0.083***	**0.046***	**0.215***	**0.373***	**0.440***	1.000	
	♂	**0.028***	**-0.025**	**0.057***	**0.027***	**0.057***	**0.035***	**0.182***	**0.402***	**0.447***	1.000	
obsessions	♀	**0.067***	**0.062***	**0.052***	**0.073***	**0.098***	**0.081***	**0.228***	**0.420***	**0.385***	**0.480***	1.000
	♂	**0.047***	0.009	**0.074***	**0.043***	**0.078***	**0.083***	**0.226***	**0.452***	**0.410***	**0.472***	1.000

Supplementary table S2. Cross-correlations between source variables of the sickness index. This table shows Kendall's Taus for associations for women (♀) and men (♂). Associations with p values under 0.05 are in bold and those with p values under 0.005 are marked with asterisks.

		age		socioeconomic status	current partnership	height	size of place of residence	education	church membership
		N	Kendall’s Tau						
age at menarche	♀	10,123	**0.081***	**0.016**	0.001	**0.089***	-0.006	**0.029***	0.001
age at first sexual intercourse	♀	10,166	**0.167***	**0.020***	**-0.035***	**0.032***	0.005	**0.212***	**0.089***
	♂	11,681	**0.129***	0.010	**-0.025***	-0.004	**-0.036***	**0.199***	**0.062***
sexual desire	♀	10,448	**-0.103***	**-0.039***	**-0.221***	**0.017**	**0.014**	**-0.031***	**-0.060***
	♂	12,150	**-0.083***	**-0.016**	**-0.097***	0.007	**0.013**	**-0.021***	**-0.033***
sexual activity	♀	10,426	**-0.059***	**0.028***	**0.367***	**0.025***	**-0.020***	-0.004	**-0.019**
	♂	12,180	**0.024***	**0.070***	**0.379***	**0.042***	0.005	**0.016**	**-0.027***
number of sexual partners	♀	9,412	**-0.056***	**-0.019**	**-0.047***	**0.025***	**0.028***	-0.003	**-0.072***
	♂	12,111	**0.022***	**0.043***	**0.081***	**0.028***	**0.022***	**0.016**	**-0.051***
number of children	♀	8,499	**0.587***	0.009	**0.104***	**-0.038***	**-0.016**	**-0.133***	**0.069***
	♂	13,435	**0.558***	**0.076***	**0.242***	**-0.018***	-0.007	**0.030***	**0.073***

Supplementary table S3. Associations of age and potential covariates with variables related to sexual life and reproduction. Columns 3 and 4 display numbers (N) of women (♀) and men (♂) and Kendall’s Taus for associations with age. Columns to the right then show partial Kendall’s Taus (age controlled). Current partnership and church membership were treated as binary variables with 0 indicating no current partnership and no church membership, and 1 indicating being in a partnership and church membership. Socioeconomic status, height, size of place of residence, and education were semi-continuous or ordinal variables where a higher value means a higher level reached in the given variable. Associations with p values under 0.05 are in bold and those with p values under 0.005 are marked with asterisks.

	women												
	age	socio-economic status	current partner-ship	height	size of place of residence	education	church member-ship	sickness index	Rh-negativity	sickness-age interaction	Rh-age interaction	sickness-Rh interaction	sickness-age-Rh interaction
age at menarche	**0.136***			**0.107***		0.023		**-0.082***	0.031	-0.056	-0.052	-0.018	0.109
age at first sexual intercourse	**0.103***		**-0.143***	**0.032**		**0.237***	**0.381***	-0.013	0.004	0.039	0.001	0.005	0.046
sexual desire	**-0.170***	-0.015	**-0.494***			-0.010	**-0.173***	**0.119***	-0.020	**-0.060**	0.014	-0.017	-0.008
sexual activity	**-0.168***	-0.011	**0.921***	0.020				**-0.086***	-0.017	**-0.106***	-0.024	-0.005	-0.041
number of sexual partners	**-0.135***		-0.008	**0.025**	0.016		**-0.195***	-0.023	-0.019	**-0.119***	0.055	0.045	-0.058
number of children	**0.681***		**0.330***	0.005		**-0.058***	**0.146***	**-0.067***	0.013	**-0.088***	0.033	-0.037	-0.004
	men												
	age	socio-economic status	current partner-ship	height	size of place of residence	education	church member-ship	sickness index	Rh-negativity	sickness-age interaction	Rh-age interaction	sickness-Rh interaction	sickness-age-Rh interaction
age at first sexual intercourse	**0.100***		**-0.153***		**-0.046***	**0.196***	**0.248***	**0.070**	**-0.084**	0.010	-0.040	-0.071	0.002
sexual desire	**-0.151***		**-0.224***			-0.002	**-0.131***	**0.145***	0.023	**-0.062**	0.047	-0.060	**0.157***
sexual activity	**-0.101***	0.010	**0.935***	0.005			**-0.126***	**-0.164***	0.041	**-0.052**	0.034	-0.011	0.083
number of sexual partners	-0.016	**0.036***	**0.180***	0.016	0.018		**0.200***	-0.035	**0.070**	**-0.058**	-0.056	0.011	0.075
number of children	**0.607***	0.019	**0.489***		**-0.026***	0.001	**0.171***	-0.021	0.042	**-0.093***	0.030	-0.063	-0.051

Supplementary table S4. Associations of Rh-negativity and covariates with each variable related to sexual life and reproduction. This table shows the results (standardised beta coefficients) of eleven GLM analyses (Type III sums of squares) which analyse associations between variables related to sexual life and reproduction (first column) and Rh-negativity, the sickness index, and covariates. In accordance with preregistration, we included in all models only covariates with Kendall’s Tau > 0.02 or < -0.02 for correlation with the output variables (see Supplementary table S3). Current partnership, church membership, and Rh-negativity were treated as binary variables with 0 indicating no current partnership, no church membership, and having a positive Rh phenotype, and 1 indicating being in a partnership, church membership, and having a negative Rh phenotype. Associations with p values under 0.05 are in bold and those with p values under 0.005 are marked with asterisks.

	women								
	age	socioeconomic status	current partnership	height	size of place of residence	education	church membership	sickness index	sickness index-age interaction
age at menarche	**0.115***			**0.116***		**0.018**		**-0.093***	-0.013
age at first sexual intercourse	**0.122***		**-0.109***	**0.028***		**0.250***	**0.339***	0.004	0.038
sexual desire	**-0.155***	**-0.013***	**-0.563***			0.003	**-0.190***	**0.125***	**-0.056***
sexual activity	**-0.165***	-0.008	**0.963***	**0.025***				**-0.096***	**-0.098***
number of sexual partners	**-0.110***		-0.047	**0.033***	**0.027**		**-0.209***	-0.044	**-0.106***
number of children	**0.708***		**0.286***	0.002		**-0.070***	**0.128***	**-0.076***	**-0.077***
	men								
	age	socioeconomic status	current partnership	height	size of place of residence	education	church membership	sickness index	sickness index-age interaction
age at first sexual intercourse	**0.106***		**-0.135***		**-0.045***	**0.207***	**0.188***	**0.062***	0.014
sexual desire	**-0.121***		**-0.218***			0.012	**-0.099***	**0.140***	**-0.054***
sexual activity	**-0.090***	**0.019**	**1.005***	**0.023***			**-0.107***	**-0.158***	**-0.043**
number of sexual partners	-0.015	**0.038***	**0.211***	**0.020**	**0.017**		**-0.145***	**-0.058***	-0.024
number of children	**0.628***	**0.022***	**0.422***		**-0.020***	**-0.014**	**0.162***	**-0.041***	**-0.109***

Supplementary table S5. Associations of the sickness index and covariates with each variable related to sexual life and reproduction in GLM analyses using Type I sums of squares. This table shows the results (standardised beta coefficients) of eleven univariate GLM analyses which analyse associations between variables related to sexual life and reproduction (first column) and the sickness index and covariates. In accordance with preregistration, each model includes only covariates with Kendall's Tau > 0.02 or < -0.02 for correlation with output variables (see Supplementary table S3). Current partnership and church membership were treated as binary variables with 0 indicating no current partnership and no church membership, and 1 indicating being in a partnership and church membership. Associations with p values under 0.05 are in bold and those with p values under 0.005 are marked with asterisks.

	women								
	age	socioeconomic status	current partnership	height	size of place of residence	education	church membership	sickness index	sickness index-age interaction
age at menarche	**0.115***			**0.123***		0.020		**-0.094***	-0.014
age at first sexual intercourse	**0.122***		**-0.109***	**0.030***		**0.277***	**0.339***	0.005	0.038
sexual desire	**-0.159***	-0.013	**-0.569***			0.001	**-0.185***	**0.130***	**-0.052**
sexual activity	**-0.172***	-0.007	**0.962***	**0.024**				**-0.091***	**-0.096***
number of sexual partners	**-0.110***		-0.046	**0.035***	**0.028**		**-0.203***	**-0.045**	**-0.106***
number of children	**0.707***		**0.287***	0.002		**-0.077***	**0.132***	**-0.075***	**-0.078***
	men								
	age	socioeconomic status	current partnership	height	size of place of residence	education	church membership	sickness index	sickness index-age interaction
age at first sexual intercourse	**0.107***		**-0.138***		**-0.050***	**0.242***	**0.189***	**0.061***	0.015
sexual desire	**-0.122***		**-0.217***			0.014	**-0.099***	**0.140***	**-0.054***
sexual activity	**-0.094***	**0.022**	**1.002***	**0.024**			**-0.103***	**-0.158***	**-0.043**
number of sexual partners	-0.016	**0.045**	**0.208***	**0.021**	**0.020**		**-0.141***	**-0.053**	-0.031
number of children	**0,628***	**0,024***	**0,424***		**-0,020***	**-0,014**	**0,165***	**-0,040***	**-0,109***

Supplementary table S6. Associations of the sickness index and covariates with each variable related to sexual life and reproduction in GLM analyses with complete data. This table shows the results (standardised beta coefficients) of eleven GLM analyses (Type III sums of squares) which analyse associations between variables related to sexual life and reproduction (first column) and the sickness index and covariates. In accordance with preregistration, each model includes only covariates with Kendall's Tau > 0.02 or < -0.02 for correlation with output variables (see Supplementary table S3). Current partnership and church membership were treated as binary variables with 0 indicating no current partnership and no church membership, and 1 indicating being in a partnership and church membership. Associations with p values under 0.05 are in bold and those with p values under 0.005 are marked with asterisks.

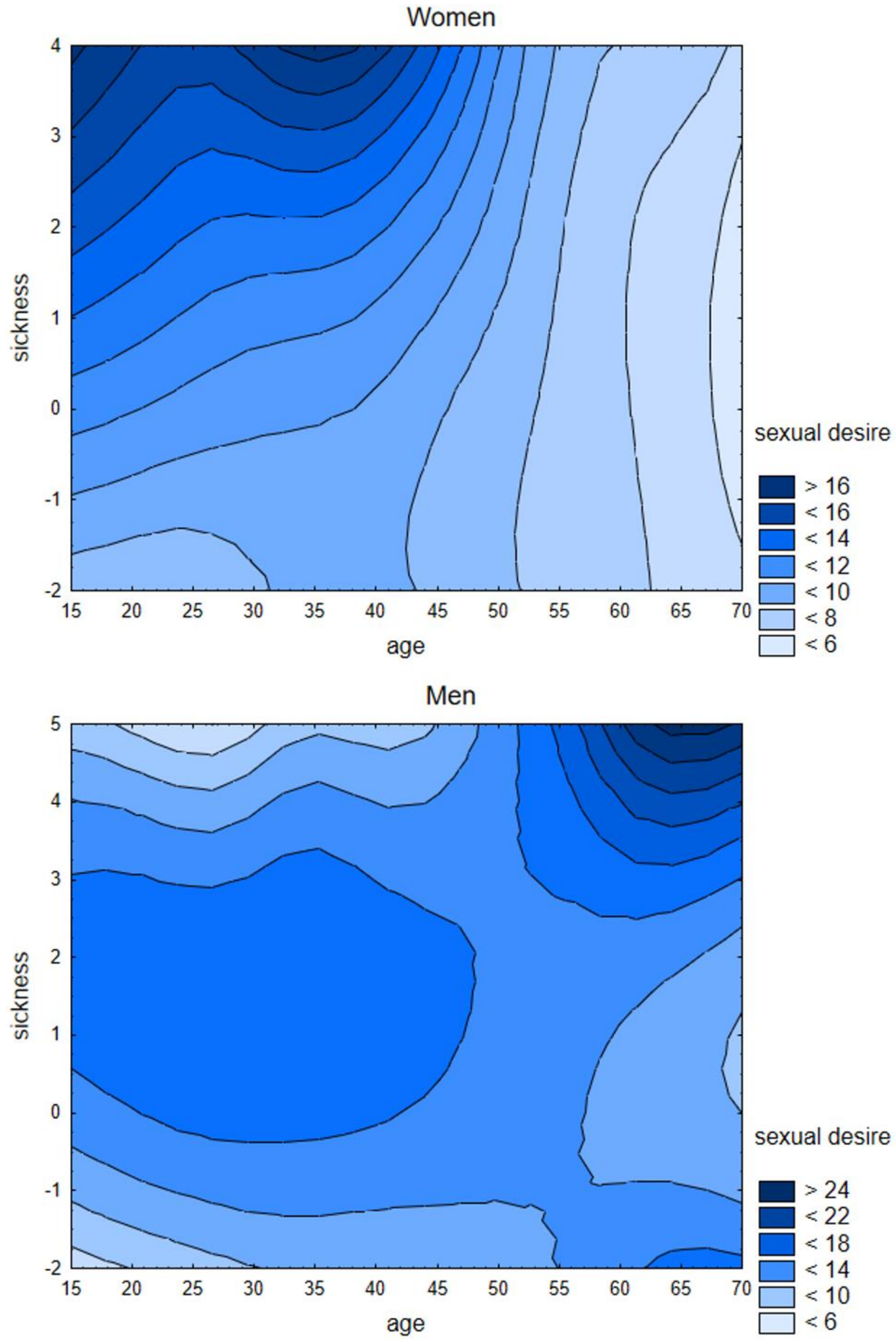

Supplementary figure S1. The effects of the sickness index and age on sexual desire in women and men. This figure shows XYZ contour plots with distance-weighted least-squares fitting used for extrapolation.

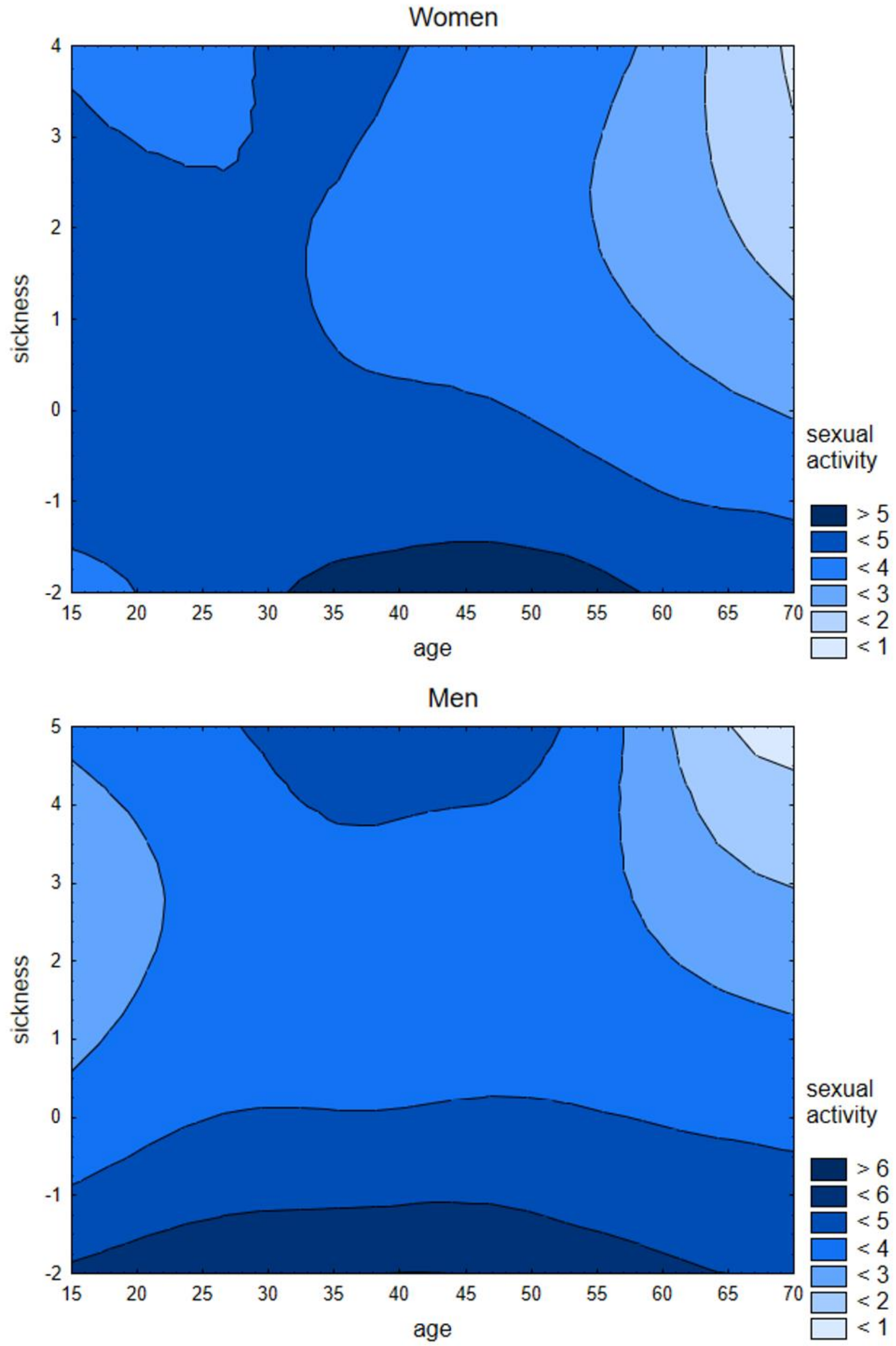

Supplementary figure S2. The effects of the sickness index and age on sexual activity in women and men. This figure shows XYZ contour plots with distance-weighted least-squares fitting used for extrapolation.

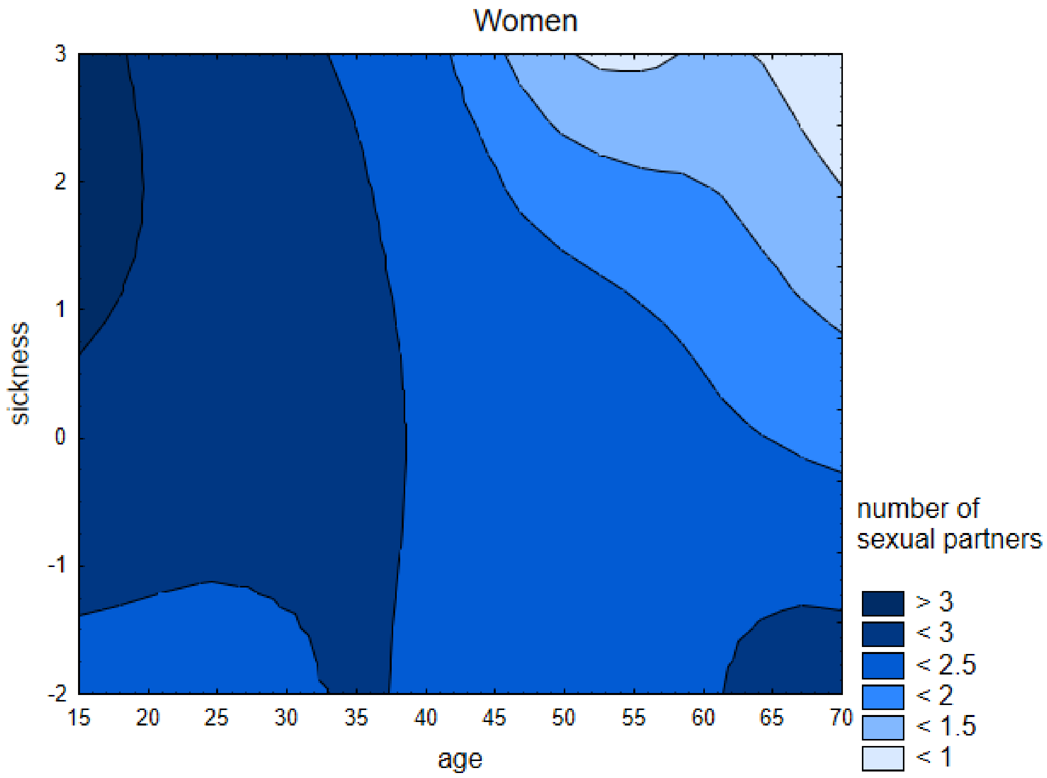

Supplementary figure S3. The effects of the sickness index and age on the number of sexual partners in women. This figure shows XYZ contour plots with distance-weighted least-squares fitting used for extrapolation.

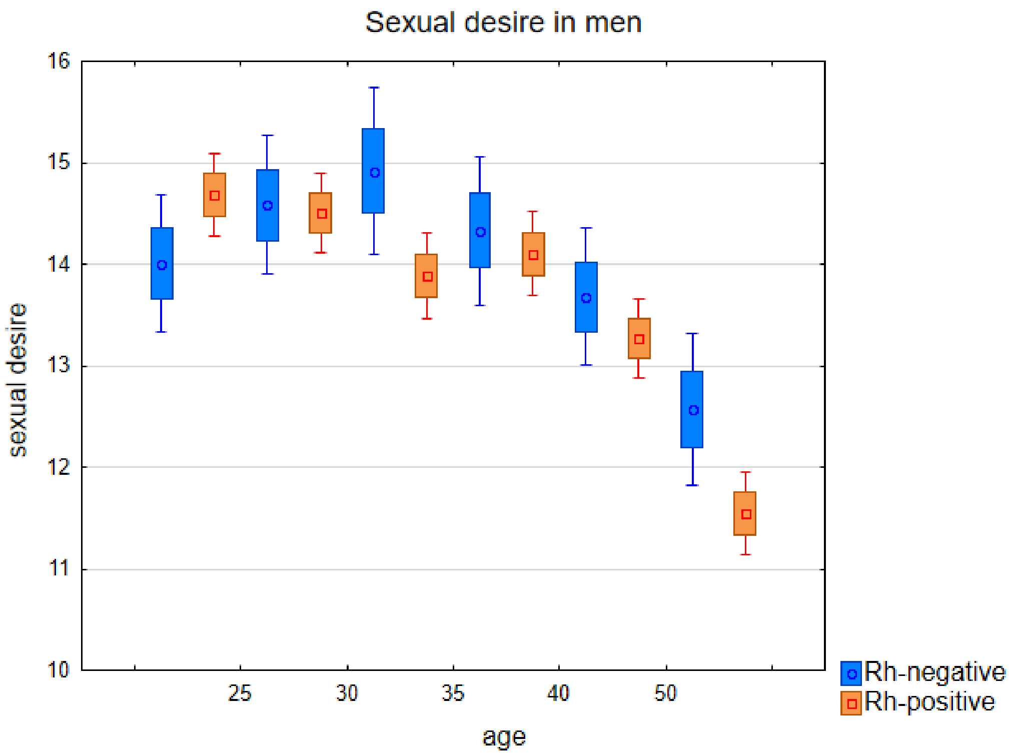

Supplementary figure S4. Box plot of sexual desire in men. This figure shows a box plot of sexual desire against age and Rh phenotype for men. Middle point = mean; box = mean+SE; whiskers = mean+95 confidence interval.

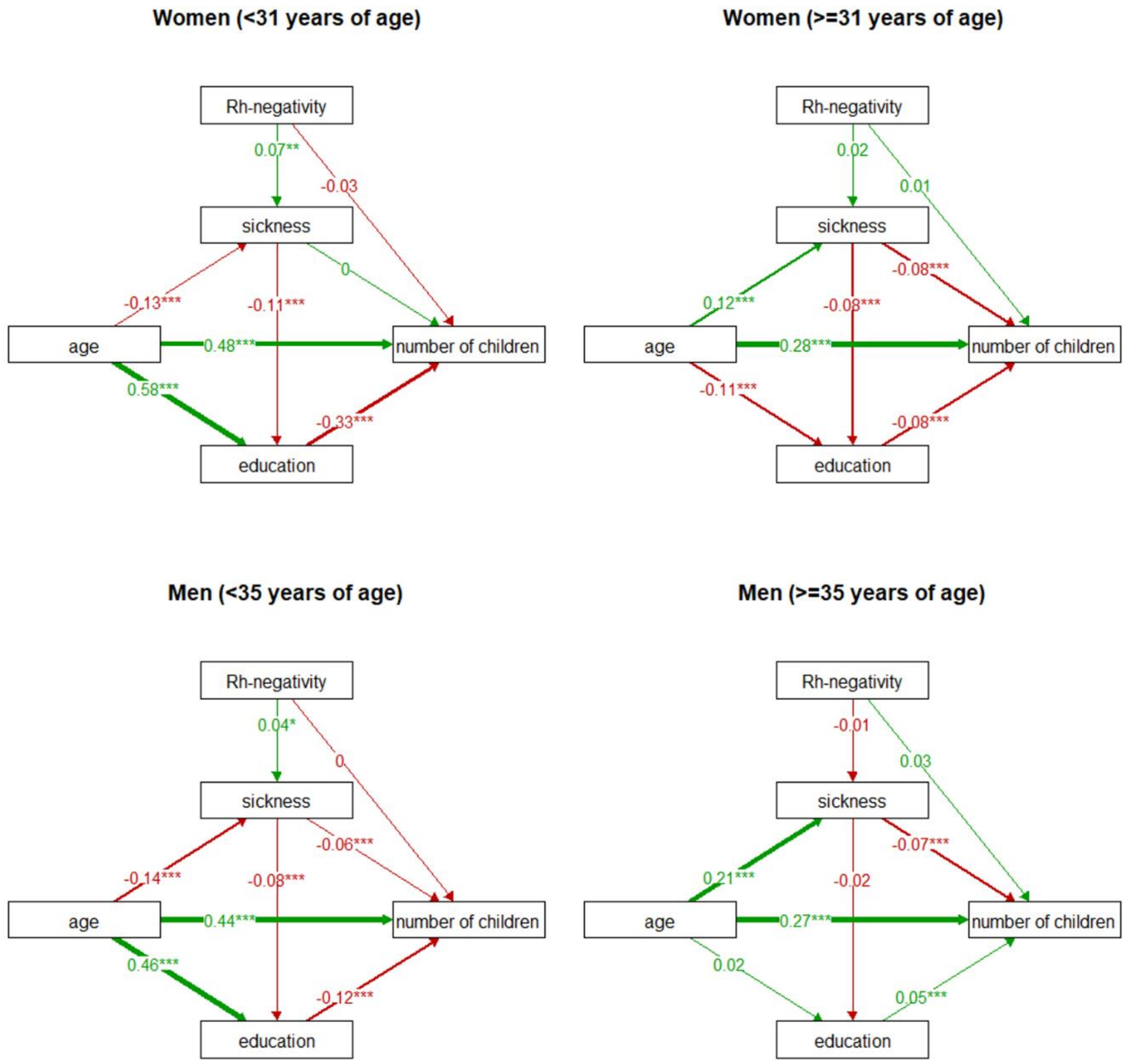

Supplementary figure S5. Direct and indirect effects of Rh-negativity on the number of children in women and men. This figure displays the results of path analyses showing the relations between the number of children, Rh-negativity, the sickness index, education, and age for women under 31 years of age (top left), women over 31 years of age (top right), men under 35 years of age (bottom left), and men over 35 years of age (bottom right). The numbers at arrows show standardised parameter estimates. Associations with p values under 0.01 are marked by '**', and those with p values under 0.001 are marked by '***'.
